# Characteristics of autoantibody-positive individuals without high-risk HLA-DR4-DQ8 or HLA-DR3-DQ2 haplotypes

**DOI:** 10.1007/s00125-024-06338-7

**Published:** 2024-12-13

**Authors:** Maria J. Redondo, David Cuthbertson, Andrea K. Steck, Kevan C. Herold, Richard Oram, Mark Atkinson, Todd M. Brusko, Hemang M. Parikh, Jeffrey P. Krischer, Suna Onengut-Gumuscu, Stephen S. Rich, Jay M. Sosenko, the Type 1 Diabetes TrialNet Study Group

**Affiliations:** 1 Texas Children’s Hospital, Department of Pediatrics, Baylor College of Medicine, Houston, TX USA; 2 Health Informatics Institute, University of South Florida, Tampa, FL USA; 3 Barbara Davis Center for Diabetes, University of Colorado Anschutz Medical Campus, Aurora, CO USA; 4 Immunobiology and Internal Medicine (Endocrinology), Yale University, New Haven, CT USA; 5 Clinical and Biomedical Sciences, University of Exeter, Exeter, UK; 6 Departments of Pathology and Pediatrics, Diabetes Institute, College of Medicine, University of Florida, Gainesville, FL USA; 7 Department of Genome Sciences, University of Virginia, Charlottesville, VA USA; 8 University of Miami Miller School of Medicine, University of Miami, Miami, FL USA

**Keywords:** Autoantibodies, Diagnosis, Endotype, Genetics, Genotype, Heterogeneity, HLA, Immunologic, Phenotype, Precision medicine, Preclinical, Prediction, Screening, TrialNet, Type 1 diabetes

## Abstract

**Aims/hypothesis:**

Many studies of type 1 diabetes pathogenesis focus on individuals with high-risk HLA haplotypes. We tested the hypothesis that, among islet autoantibody-positive individuals, lacking HLA-DRB1*04-DQA1*03-DQB1*0302 (HLA-DR4-DQ8) and/or HLA-DRB1*0301-DQA1*0501-DQB1*0201 (HLA-DR3-DQ2) is associated with phenotypic differences, compared with those who have these high-risk HLA haplotypes.

**Methods:**

We classified autoantibody-positive relatives of individuals with type 1 diabetes into four groups based on having both HLA-DR4-DQ8 and HLA-DR3-DQ2 (DR3/DR4; *n*=1263), HLA-DR4-DQ8 but not HLA-DR3-DQ2 (DR4/non-DR3; *n*=2340), HLA-DR3-DQ2 but not HLA-DR4-DQ8 (DR3/non-DR4; *n*=1607) and neither HLA-DR3-DQ2 nor HLA-DR4-DQ8 (DRX/DRX; *n*=1294). Group comparisons included demographics, metabolic markers and the prevalence of autoantibodies against GAD65 (GADA%), IA-2 (IA-2A%) or insulin (IAA%) at enrolment. A *p* value <0.01 was considered statistically significant.

**Results:**

IA-2A% was lower in the DRX/DRX group (20.9%) than in the DR4/non-DR3 (38.5%, *p*<0.001) and DR3/DR4 (44.8%, *p*<0.001) groups, but similar to the DR3/non-DR4 group (20.0%). Conversely, IAA% was similar in the DRX/DRX (43.4%), DR4/non-DR3 (41.1%) and DR3/DR4 (41.0%) groups, but lower in the DR3/non-DR4 group (30.1%, *p*<0.001). Participants in the DRX/DRX group were older, with a lower prevalence of White participants and a higher prevalence of overweight/obesity, and higher preserved C-peptide (as measured by a lower Index60) than those in the DR3/DR4 group (all comparisons, *p*<0.005), a lower prevalence of White or non-Hispanic participants and a lower Index60 than those in the DR4/non-DR3 group, and younger age, a higher prevalence of Hispanic participants and a lower Index60 than those in the DR3/non-DR4 group (all comparisons, *p*<0.005). Among the 1292 participants who progressed to clinical type 1 diabetes, those in the DR3/non-DR4 group had higher GADA%, lower IA-2A% and lower IAA% than the other groups (all comparisons, *p*<0.01), and those in the DR3/DR4 group had the youngest age at diagnosis (all comparisons, *p*<0.001).

**Conclusions/interpretation:**

Autoantibody-positive individuals who lack both high-risk HLA haplotypes (DRX/DRX) or have HLA-DR3-DQ2 but lack HLA-DR4-DQ8 (DR3/non-DR4) have phenotypic differences compared with DR3/DR4 and DR4/non-DR3 individuals, suggesting that there is aetiological heterogeneity in type 1 diabetes.

**Graphical Abstract:**

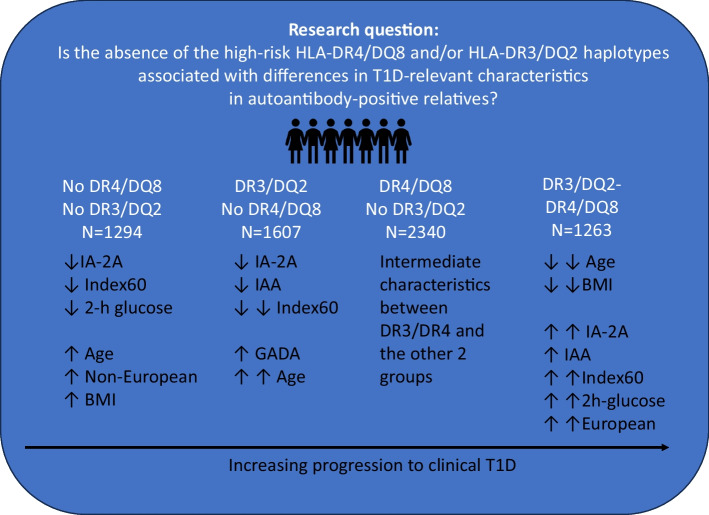

**Supplementary Information:**

The online version of this article (10.1007/s00125-024-06338-7) contains peer-reviewed but unedited supplementary material.


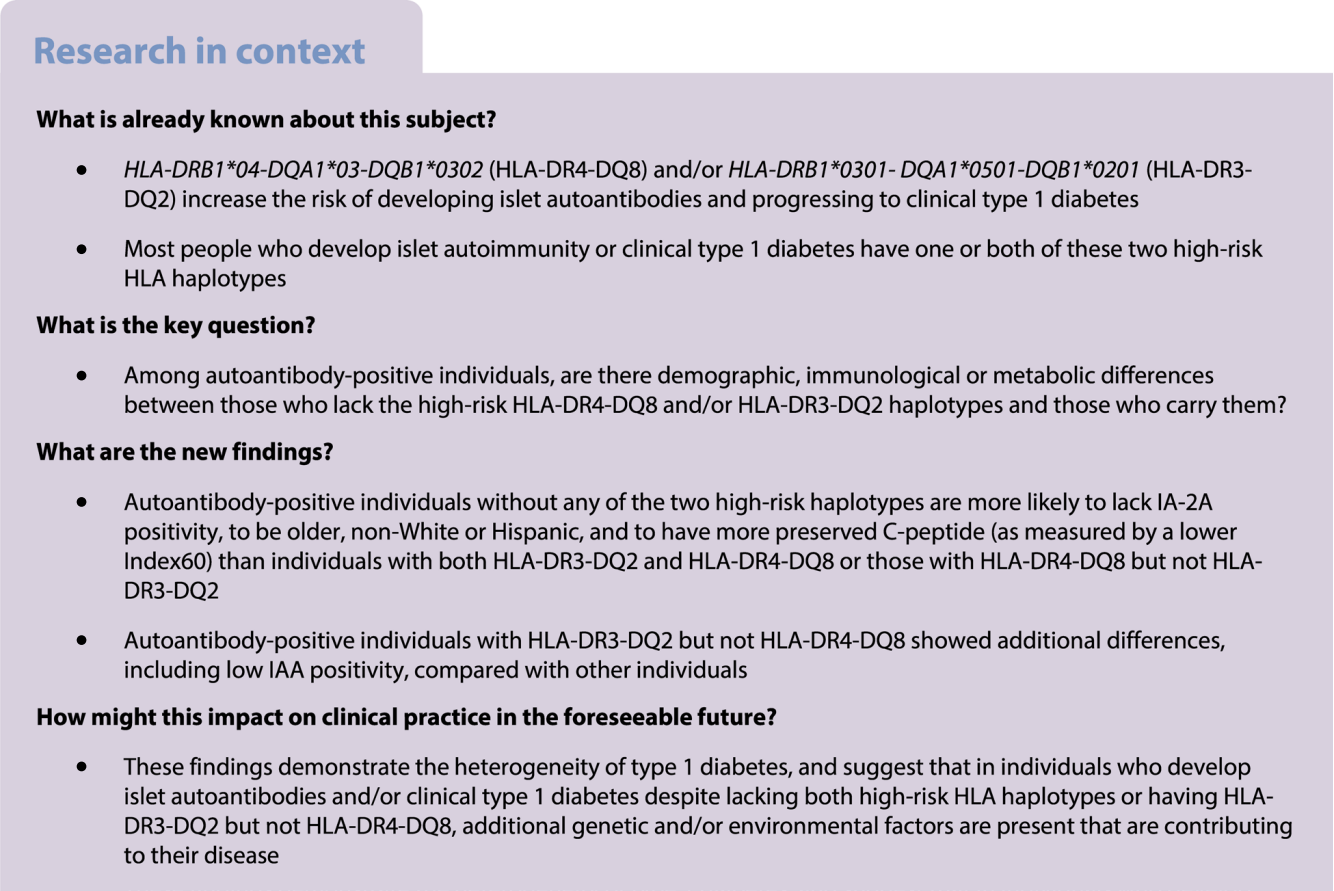


## Introduction

Type 1 diabetes is a life-threatening, chronic disease resulting from T cell-mediated autoimmunity against insulin-producing beta cells in the pancreas [1]. Decades of research have produced an accumulation of knowledge on the pathogenesis of type 1 diabetes, which has translated into accurate predictive models [2], and, more recently, therapies that can delay the clinical onset of the disease [3] and the loss of beta cell function that continues after diagnosis [4–6].

Due to the relatively low frequency of type 1 diabetes in the general population, many prospective studies on the natural history of type 1 diabetes have followed children with increased a priori risk of the disease based on the presence of specific HLA haplotypes that are strongly associated with the development of type 1 diabetes [7, 8]. While this study design increases the feasibility of studies and provides important knowledge, it excludes the sizeable proportion of people who develop the disease despite the absence of high-risk HLA haplotypes. Furthermore, as most of the classical genetic associations were discovered in research cohorts of predominantly White European participants diagnosed with typical paediatric-onset type 1 diabetes, studies of individuals without the high-risk HLA haplotypes should provide a broader perspective of the type 1 diabetes spectrum in terms of age, genetic ancestries and other features.

Therefore, we tested the hypothesis that islet autoantibody-positive individuals who lack HLA-DRB1*04-DQA1*03-DQB1*0302 (DR4-DQ8) and/or DRB1*03:01-DQB1*05:01-DQB1*02:01 (DR3-DQ2) differ from those who carry these two high-risk haplotypes in terms of characteristics that are known to predict the progression to type 1 diabetes. This knowledge should ultimately increase our understanding of the pathogenesis of type 1 diabetes in diverse cohorts of individuals, which may lead to improvements in our ability to predict type 1 diabetes and in personalised strategies to prevent its progression.

## Methods

### Participants

Since 2004, the TrialNet Pathway to Prevention study has screened for islet autoantibodies in more than 230,000 non-diabetic relatives of patients with type 1 diabetes [9]. All TrialNet Pathway to Prevention participants were tested for islet autoantibodies to GAD65 (GADA), insulin (IAA) and insulinoma-associated antigen-2 (IA-2A), as previously described [9, 10]. Participants who were found to be autoantibody-positive underwent HLA genotyping and an OGTT, with glucose and C-peptide measurements, as previously reported [9, 11]. HLA genotyping was performed at the TrialNet Core Laboratory at the Barbara Davis Center for Diabetes (Aurora, CO, USA), and genome-wide genotyping was performed using the T1DExomeChip (Illumina, USA) at the University of Virginia (Charlottesville, VA, USA), as previously described [12, 13]. All study participants (or their parents/guardians if appropriate) provided written informed consent, and children provided assent as appropriate, prior to screening and enrolment, and the study was approved by the responsible ethics committee at each TrialNet site. Eligibility criteria for this analysis included being a participant in the TrialNet Pathway to Prevention study and having at least one positive islet autoantibody and carrying: (1) neither HLA-DR3-DQ2 nor DR4-DQ8 (referred to as DRX/DRX) (*n*=1294); (2) HLA-DR3-DQ2/DR4-DQ8 (referred to as DR3/DR4) (*n*=1263); (3) HLA-DR4-DQ8 but not DR3-DQ2 (referred to as DR4/non-DR3) (*n*=2340); and (4) HLA-DR3-DQ2 but not DR4-DQ8 (referred to as DR3/non-DR4) (*n*=1607). A total of 6504 participants were included. A flowchart that illustrates the selection of participants for this analysis is presented in electronic supplementary material (ESM) Fig. 1.

### Metabolic and genetic risk markers

We analysed available metabolic measures and genetic risk markers that are known predictors of progression to type 1 diabetes and were available to us, such as islet autoantibodies, age, self-reported gender, race, genetics, family history of type 1 diabetes, BMI and metabolic measures [2, 14–16]. We calculated Index60 using fasting C-peptide, as well as C-peptide and glucose at 60 minutes in an oral glucose tolerance test, as previously described [16]. A high Index60 has been shown to be associated with characteristics that are typical of type 1 diabetes and high-risk of progression to clinical disease [16, 17]. Dysglycaemia was defined as OGTT values of 6.1–6.9 mmol/l when fasting, ≥11.1 mmol/l at 30, 60 and/or 90 min, and/or 7.8–11.0 mmol/l at 2 h, as previously described [18]. Obesity was defined as BMI ≥30 kg/m^2^ for adults [19] or ≥95th age- and sex-adjusted percentile for children [20]. Overweight was defined as BMI ≥25 and <30 kg/m^2^ for adults [19] or ≥85th and <95th age- and sex-adjusted percentile for children [20]. The type 1 diabetes genetic risk score 2 (T1D-GRS2), which combines 67 SNPs in HLA and non-HLA genetic regions that are associated with type 1 diabetes, was calculated as previously described [13, 21, 22]. In brief, we added the number of risk-increasing alleles at each SNP position (0, 1 or 2), multiplied by the amount that the SNP increases the risk of type 1 diabetes. Several HLA class II alleles showed strong interactions as we included specific interaction terms for 18 HLA DQ allele combinations in the score. In addition, we calculated the HLA and non-HLA components of the T1D-GRS2 using 35 SNPs tagging HLA alleles and the remaining 32 SNPs, located outside the HLA region, respectively.

### Statistical analysis

We compared autoantibody-positive DRX/DRX relatives with participants in the DR4/non-DR3, DR3/DR4 and DR3/non-DR4 groups using ANOVA for continuous variables, and χ^2^ tests or the Fisher exact test for categorical variables, as appropriate. Group comparisons included the frequency of positivity for GADA, IA-2A and IAA, Index60 and demographic information. These comparisons were performed both overall and pairwise between HLA groups. Additionally, the comparisons were confirmed using age-adjusted general linear models for continuous parameters, and categorical data models. General linear models were used to test for interactions between HLA status and participant characteristics for each of the metabolic measures. Kaplan–Meier and age-adjusted Cox regression analyses were used to test for HLA group differences related to progression to type 1 diabetes. All analyses were performed using SAS version 9.4 (SAS Institute, USA). To account for multiple testing, based on a Bonferroni adjustment, for unrelated pairwise comparisons between HLA categories, *p* values <0.01 were considered statistically significant.

## Results

### Comparisons among relatives who were autoantibody-positive and had no clinical type 1 diabetes at enrolment

We first compared characteristics between the DRX/DRX group and each of the other three genetic groups (i.e. DR3/DR4, DR4/non-DR3 and DR3/non-DR4) in initially non-diabetic TrialNet participants (Table 1). The prevalence of positivity for IA-2A in the DRX/DRX group (20.9%) was approximately half that in the DR3/DR4 group (44.8%, *p*<0.001) or the DR4/non-DR3 group (38.5%, *p*<0.001), but there was no evidence of a difference between the DRX/DRX and DR3/non-DR4 groups. The IAA prevalence in the DRX/DRX group (43.4%) was higher than in the DR3/non-DR4 group (30.1%, *p*<0.001) but was not statistically different from that in the DR3/DR4 or DR4/non-DR3 groups. The GADA prevalence in the DRX/DRX group (76.0%) was significantly lower than that in the DR3/DR4 group (84.5%, *p*<0.001) and the DR3/non-DR4 group (83.6%, *p*<0.001), but was not significantly different compared with the DR4/non-DR3 group.

**Table 1:** Characteristics of autoantibody-positive relatives of individuals with type 1 diabetes

Parameter	DRX/DRX (A) (*N*=1294)	DR3/NON-DR4 (B) (*N*=16,070	DR4/NON-DR3 (C) (*N*=2340)	DR3/DR4 (D) (*N*=1263)	Overall *p* value	*p* value (A vs B)	*p* value (A vs C)	*p* value (A vs D)	*p* value (B vs C)	*p* value (B vs D)	*p* value (C vs D)
GADA					<0.001***	<0.001***	0.012**	<0.001***	0.002**	0.510	<0.001***
Negative	24.0 (311)	16.4 (264)	20.4 (478)	15.5 (196)							
Positive	76.0 (983)	83.6 (1343)	79.6 (1862)	84.5 (1067)							
IA-2A					<0.001***	0.520	<0.001***	<0.001***	<0.001***	<0.001***	<0.001
Negative	79.1 (1023)	80.0 (1286)	61.5 (1440)	55.2 (697)							
Positive	20.9 (271)	20.0 (321)	38.5 (900)	44.8 (566)							
IAA					<0.001***	<0.001***	0.167	0.216**	<0.001***	<0.001**	0.974
Negative	56.6 (732)	69.9 (1124)	58.9 (1379)	59.0 (745)							
Positive	43.4 (562)	30.1 (483)	41.1 (961)	41.0 (518)							
Age (years) at autoantibody draw	17.06 ± 12.67 (1294)	18.48 ± 13.72 (1605)	16.92 ± 12.91 (2338)	13.96 ± 11.30 (1263)	<0.001	0.004	0.746	<0.001	<0.001	<0.001	<0.001
Gender					0.120	0.466	0.982	0.119	0.414	0.018	0.074
Female	52.0 (672)	53.3 (856)	52.0 (1216)	48.9 (617)							
Male	48.0 (621)	46.7 (749)	48.0 (1122)	51.1 (645)							
Race					<0.001***	0.390	<0.001***	<0.001***	<0.001**	<0.001**	0.682
Asian	2.5 (32)	2.1 (34)	0.9 (20)	1.0 (13)							
Black or African American	4.4 (56)	4.5 (72)	2.4 (56)	1.8 (23)							
Multiracial	2.3 (29)	1.5 (24)	1.7 (39)	1.2 (15)							
White	83.0 (1058)	85.2 (1353)	87.8 (2026)	89.1 (1110)							
Refused	0.4 (5)	0.2 (3)	0.2 (5)	0.2 (3)							
Unknown^a^	7.5 (95)	6.4 (102)	7.0 (161)	6.6 (82)							
Hispanic or Latino^b^					<0.001***	<0.001***	0.008**	0.027	0.014	0.067	0.895
Yes	13.5 (174)	7.7 (123)	10.2 (237)	10.1 (127)							
No	82.5 (1060)	88.2 (1409)	85.1 (1970)	85.5 (1074)							
Unknown^a^	4.0 (51)	4.1 (65)	4.7 (109)	4.4 (55)							
Overweight or obese					<0.001	0.083	0.230	0.004	<0.001	<0.001	0.041
No	75.3 (945)	72.4 (1128)	77.1 (1763)	80.1 (981)							
Yes	24.7 (310)	27.6 (430)	22.9 (524)	19.9 (244)							
Baseline Index60	−0.17 ± 1.29 (1294)	0.02 ± 1.36 (1607)	0.10 ± 1.45 (2340)	0.38 ± 1.38 (1263)	<0.001***	<0.001***	<0.001***	<0.001***	0.099	<0.001***	<0.001***
T1D-GRS2	11.05 ± 2.07 (792)	12.83 ± 1.90 (990)	13.59 ± 1.46 (1596)	15.38 ± 1.12 (920)	<0.001***	<0.001***	<0.001***	<0.001***	<0.001***	<0.001***	<0.001***
HLA T1D-GRS2	7.10 ± 1.81 (792)	8.86 ± 1.74 (990)	9.58 ± 1.25 (1596)	11.47 ± 0.83 (920)	<0.001	<0.001	<0.001***	<0.001***	<0.001***	<0.001***	<0.001***
Non-HLA T1D-GRS2	3.98 ± 0.83 (792)	4.00 ± 0.84 (990)	4.01 ± 0.78 (1596)	3.92 ± 0.78 (920)	0.055*	0.653	0.364	0.138	0.676	0.042	0.006**
Relative with diabetes					<0.001***	<0.001***	<0.001**	<0.001***	<0.001	<0.001***	<0.001***
Father	9.0 (117)	11.2 (180)	11.8 (277)	8.1 (102)							
Mother	8.1 (105)	8.4 (135)	7.6 (179)	8.2 (104)							
Offspring	18.9 (245)	24.3 (391)	18.8 (440)	11.9 (150)							
Sibling	50.5 (653)	48.4 (777)	52.5 (1229)	64.4 (814)							
Other	13.4 (174)	7.7 (124)	9.2 (215)	7.4 (93)							
T1D progressor					<0.001***	<0.001***	<0.001***	<0.001***	<0.001***	<0.001***	<0.001**
No	89.0 (1152)	83.8 (1347)	77.9 (1822)	70.5 (891)							
Yes	11.0 (142)	16.2 (260)	22.1 (518)	29.5 (372)							
Follow-up duration (years)	3.06 ± 3.19 (1294)	2.89 ± 3.14 (1607)	3.03 ± 3.13 (2340)	2.85 ± 2.86 (1263)	0.166	0.153	0.799	0.075	0.165	0.697	0.081
Fasting glucose (mmol/l)	5.11 ± 0.73 (1294)	5.16 ± 0.70 (1607)	5.17 ± 1.18 (2340)	5.09 ± 0.72 (1263)	0.028	0.076	0.079	0.464	0.630	0.011	0.021
2 h glucose (mmol/l)	6.59 ± 2.46 (1294)	6.88 ± 2.55 (1607)	7.07 ± 2.88 (2340)	7.25 ± 2.92 (1263)	<0.001***	0.002**	<0.001***	<0.001***	0.031	<0.001**	0.076
Dysglycaemia					<0.001***	0.001***	<0.001***	<0.001***	0.077	<0.001**	0.028*
No	80.0 ± 1035)	73.6 (1182)	71.0 (1661)	67.5 (852)							
Yes	20.0 (259)	26.4 (425)	29.0 (679)	32.5 (411)							
HbA_1c_ (mmol/mol)	43.29 ± 0.42 (1267)	32.66 ± 0.40 (1574)	32.55 ± 0.42 (2273)	32.74 ± 0.38 (1238)	0.035*	0.040	0.106	0.004**	0.571	0.316	0.117
HbA_1c_ (%)	5.11 ± 0.04 (1267)	5.14 ± 0.04 (1574)	5.13 ± 0.04 (2273)	5.15 ± 0.03 (1238)	0.035*	0.040	0.106	0.004**	0.571	0.316	0.117

Values for categorical variables are % (*n*) and those for continuous variables are means ± SD (*n*). Denominators for calculation of the percentages are not the numbers at the top of the column due to missing dataComparison of DRX/DRX, DR3/non-DR4, DR4/non-DR3 and DR3/DR4 groups (DR4, HLA-DRB1*04-DQA1*03-DQB1*0302; DR3, HLA-DRB1*0301-DQA1*0501-DQB1*0201; DRX, HLA haplotype that is neither HLA-DRB1*04-DQA1*03-DQB1*0302 nor HLA-DRB1*0301-DQA1*0501-DQB1*0201). Characteristics are at enrolment except where noted otherwise

^a^The category ‘unknown’ represents those who reported their race as unknown, rather than missing data

^b^Hispanic/Latino is shown separately because race and ethnicity were collected as two different sets of classifications in the USA (until 2024)

Values that were statistically significant in the age-adjusted model are indicated by asterisks: *p<0.05; **p<0.01; ***p<0.001

T1D, type 1 diabetes

Overweight/obesity was more prevalent in relatives carrying DRX/DRX (24.7%) than in the group DR3/DR4 (19.9%, *p*<0.004), but it was not significantly different compared with DR4/non-DR3 or DR3/non-DR4 relatives. Index60 was significantly lower in relatives with DRX/DRX compared with those carrying DR3/DR4, DR4/non-DR3 or DR3/non-DR4 (all comparisons, *p*<0.001). There were no significant differences in fasting glucose; however, 2 h glucose during an OGTT and the prevalence of dysglycaemia were lowest in the DRX/DRX group (all comparisons, *p*≤0.002). The age at the time when participants were identified as autoantibody-positive was significantly greater in DRX/DRX relatives (mean 17.06 years) than in DR3/DR4 relatives (mean 13.96 years, *p*<0.001) but lower than in DR3/non-DR4 relatives (18.48 years, *p*<0.004). The proportion of self-reported White participants was lower in the DRX/DRX group (83.0%) than in the DR3/DR4 group (89.1%, *p*<0.001) and the DR4/non-DR3 group (87.8%, *p*<0.001), but was not statistically different from that in the DR3/non-DR4 group. The DRX/DRX group had a higher proportion of Hispanic/Latino participants (13.5%) compared with the DR4/non-DR3 group (10.2%, *p*=0.008) and the DR3/non-DR4 group (7.7%, *p*<0.001), but it did not differ statistically from the DR3/DR4 group. There were no significant differences in gender between the DRX/DRX group and any of the other HLA-defined groups.

Because of their strong association with type 1 diabetes, SNPs tagging HLA class II alleles have a large influence on the T1D-GRS2 [21], and hence this was lowest in participants in the DRX/DRX group and progressively higher in the DR3/non-DR4, DR4/non-DR3 and DR3/DR4 groups (all comparisons, *p*<0.001). The non-HLA component of the T1D-GRS2 was not statistically different between the DRX/DRX group and each of the other three genetic groups.

Comparisons between the participants with only one vs two high-risk HLA haplotypes showed that DR3/non-DR4 had more differences from DR3/DR4 than DR4/non-DR3 did (Table 1). We observed that relatives who had DR3/non-DR4 showed a lower frequency of positivity for IAA (30.1%) and IA2-A (20.0%) than those who had DR4/non-DR3 (41.1% and 38.5%, respectively) or DR3/DR4 (41.0% and 44.8%, respectively) (all comparisons, *p*<0.001). Of note, compared with DRX/DRX, the DR3/non-DR4 group had a significantly lower prevalence of IAA and a similar prevalence of IA-2A. Overweight and obesity were more frequent among DR3/non-DR4 participants (27.6%) than those in the DR4/non-DR3 (22.9%, *p*<0.001) or DR3/DR4 (19.9%, *p*<0.001) groups. Index60 was lower in the DR3/non-DR4 group than in the DR3/DR4 group (*p*<0.001). Relatives with DR3/non-DR4 were significantly older at the time of enrolment (mean 18.48 years) compared to those with DR4/non-DR3 (16.92 years, *p*<0.001) or DR3/DR4 (13.96, *p*<0.001). The distribution of type of relatives with diabetes was different among the groups, with DR3/DR4 having the highest proportion of siblings and the lowest of offspring, with and without adjustment for age (all comparisons, *p*<0.001). African American race was significantly more frequent among relatives with DR3/non-DR4 (4.5%) than those with DR4/non-DR3 (2.4%, *p*<0.001) or DR3/DR4 (1.8%, *p*<0.001).

In comparisons between the DR4/non-DR3 and DR3/DR4 groups, the former had a significantly lower prevalence of GADA and IA-2A, older age, lower Index60, lower T1D-GRS2, a lower HLA component of the T1D-GRS2 and a higher non-HLA component of the T1D-GRS. Risk of progression to clinical type 1 diabetes within the next 5 years was highest among those in the DR3/DR4 group (35.0%) and progressively lower in the DR4/non-DR3 (26.9%), DR3/non-DR4 (19.9%) and DRX/DRX groups (13.4%) (unadjusted *p*<0.001; age-adjusted *p*<0.001) (ESM Fig. 2).

### Comparisons among relatives who were autoantibody-positive and had no clinical type 1 diabetes at enrolment, but had progressed to clinical type 1 diabetes on follow-up

Among the 1292 relatives who were autoantibody-positive and had no clinical type 1 diabetes at enrolment, but had progressed to clinical type 1 diabetes on follow-up (progressors) (Table 2), those carrying DRX/DRX had a significantly lower frequency of GADA (78.9%), a higher frequency of IA-2A (65.5%) and a higher frequency of IAA (58.5%) than those with DR3/non-DR4 (91.2%, 48.1% and 41.2%, respectively; all comparisons, *p*<0.001) (Table 2). As in the full autoantibody-positive cohort, DRX/DRX group progressors were older at enrolment (mean 13.22 years) than those in the DR3/DR4 group (9.80 years, *p*<0.001), and the T1D-GRS2 was lowest in the DRX/DRX group and progressively higher in the DR3/non-DR4, DR4/non-DR3 and DR3/DR4 groups (all comparisons, *p*<0.001). Progressors with DR3/DR4 were significantly younger at the time of diagnosis (mean 12.01 years) than those in each of the other three groups (15.31, 15.69 and 14.66 years old, respectively, for the DRX/DRX, DR3/non-DR4 and DR4/non-DR3 groups; all comparisons, *p*<0.001).

**Table 2:** Characteristics of autoantibody-positive relatives who progressed to clinical type 1 diabetes (‘progressors’)

Parameter	DRX/DRX (A) (*N*=142)	DR3/non-DR4 (B) (*N*=260)	DR4/non-DR3 (C) (*N*=518)	DR3/DR4 (D) (*N*=372)	Overall *p* value	*p* value (A vs B)	*p* value (A vs C)	*p* value (A vs D)	*p* value (B vs C)	*p* value (B vs D)	*p* value (C vs D)
GADA					<0.001***	<0.001**	0.676	0.238	<0.001***	0.005**	0.025**
Negative	21.1 (30)	8.8 (23)	22.8 (118)	16.7 (62)							
Positive	78.9 (112)	91.2 (237)	77.2 (400)	83.3 (310)							
IA-2A					<0.001***	<0.001**	0.362	0.470	<0.001***	<0.001***	0.828
Negative	34.5 (49)	51.9 (135)	30.5 (158)	31.2 (116)							
Positive	65.5 (93)	48.1 (125)	69.5 (360)	68.8 (256)							
IAA					<0.001***	<0.001***	0.466	0.682	<0.001***	<0.001	0.671
Negative	41.5 (59)	58.8 (153)	45.0 (233)	43.5 (162)							
Positive	58.5 (83)	41.2 (107)	55.0 (285)	56.5 (210)							
Age (years) at autoantibody draw	13.22 ± 10.56 (142)	13.64 ± 11.36 (260)	12.56 ± 10.34 (518)	9.80 ± 7.31 (372)	<0.001	0.721	0.503	<0.001	0.187	<0.001	<0.001
Gender					0.206	0.712	0.775	0.239	0.389	0.337	0.036
Female	50.0 (71)	48.1 (125)	51.4 (266)	44.2 (164)							
Male	50.0 (71)	51.9 (135)	48.6 (252)	55.8 (207)							
Race					0.003	0.019	0.395	0.851	0.004	0.002	0.706
Asian	0.7 (1)	2.7 (7)	0.6 (3)	0.5 (2)							
Black or African American	2.1 (3)	5.9 (15)	1.9 (10)	1.6 (6)							
Multiracial	0.7 (1)	1.6 (4)	1.9 (10)	0.8 (3)							
White	92.1 (129)	82.0 (210)	89.1 (458)	91.8 (335)							
Refused	1.4 (2)	0.0 (0)	0.4 (2)	0.5 (2)							
Unknown^a^	2.9 (4)	7.8 (20)	6.0 (31)	4.7 (17)							
Hispanic or Latino^b^					0.364	0.696	0.616	0.518	0.950	0.132	0.098
Yes	7.7 (11)	7.7 (20)	8.4 (43)	11.1 (41)							
No	88.7 (126)	86.9 (225)	86.2 (442)	85.9 (318)							
Unknown^a^	3.5 (5)	5.4 (14)	5.5 (28)	3.0 (11)							
Overweight or obese					0.698*	0.496	0.510	0.981	0.916	0.355**	0.333
No	86.0 (117)	83.4 (211)	83.7 (416)	86.1 (310)							
Yes	14.0 (19)	16.6 (42)	16.3 (81)	13.9 (50)							
Baseline Index60	1.48 ± 1.44 (142)	1.41 ± 1.35 (260)	1.37 ± 1.50 (518)	1.33 ± 1.34 (372)	0.726	0.593	0.411	0.261	0.725	0.492	0.714
T1D-GRS2	12.36 ± 1.56 (110)	13.42 ± 1.65 (212)	13.90 ± 1.40 (412)	15.56 ± 1.12 (302)	<0.001***	<0.001***	<0.001***	<0.001***	<0.001***	<0.001***	<0.001***
HLA T1D-GRS2	8.14 ± 1.34 (110)	9.36 ± 1.54 (212)	9.83 ± 1.17 (412)	11.56 ± 0.84 (302)	<0.001***	<0.001***	<0.001***	<0.001***	<0.001***	<0.001***	<0.001***
Non-HLA T1D-GRS2	4.23 ± 0.72 (110)	4.07 ± 0.77 (212)	4.06 ± 0.75 (412)	4.02 ± 0.78 (302)	0.106	0.072	0.034	0.015	0.881	0.498	0.515
Relative with diabetes					<0.001	0.132	0.683	0.018	0.092	0.001	<0.001
Father	14.1 (20)	10.4 (27)	13.1 (68)	8.1 (30)							
Mother	12.7 (18)	6.5 (17)	9.7 (50)	8.6 (32)							
Offspring	7.7 (11)	11.9 (31)	8.5 (44)	3.8 (14)							
Sibling	57.7 (82)	63.5 (165)	57.7 (299)	71.8 (267)							
Other	7.7 (11)	7.7 (20)	11.0 (57)	7.8 (29)							
Age (years) at T1D diagnosis	15.31 ± 10.42 (142)	15.69 ± 11.39 (260)	14.66 ± 10.25 (518)	12.01 ± 7.40 (372)	<0.001	0.739	0.507	<0.001	0.203	<0.001	<0.001
Time to T1D diagnosis (years)	1.62 ± 1.41 (142)	1.47 ± 1.36 (260)	1.64 ± 1.49 (518)	1.76 ± 1.46 (372)	0.113	0.315	0.845	0.323	0.119	0.013	0.258
Fasting glucose (mmol/l)	5.45 ± 1.68 (142)	5.51 ± 1.19 (260)	5.39 ± 1.40 (518)	5.20 ± 1.06 (372)	0.020	0.698	0.635	0.043	0.229	<0.001	0.031
2 h glucose (mmol/l)	9.70 ± 4.86 (142)	9.66 ± 4.10 (260)	9.46 ± 4.51 (518)	9.13 ± 4.13 (372)	0.390	0.925	0.572	0.185	0.543	0.114	0.274
Dysglycaemia					0.124	0.057	0.221	0.756	0.279	0.039	0.213
No	45.1 (64)	35.4 (92)	39.4 (204)	43.5 (162)							
Yes	54.9 (78)	64.6 (168)	60.6 (314)	56.5 (210)							
HbA_1c_ (mmol/mol)	35.60 ± 0.80 (140)	35.95 ± 0.57 (255)	35.15 ± 0.61 (504)	34.43 ± 0.49 (365)	0.025	0.643	0.552	0.064	0.130	<0.001	0.063
HbA_1c_ (%)	5.41 ± 0.07 (140)	5.44 ± 0.05 (255)	5.37 ± 0.06 (504)	5.30 ± 0.04 (365)	0.025	0.643	0.552	0.064	0.130	<0.001	0.063

Values for categorical variables are % (*n*) and those for continuous variables are means ± SD (*n*). Denominators for calculation of the percentages are not the numbers at the top of the column due to missing data. Comparison of DRX/DRX, DR3/non-DR4, DR4/non-DR3 and DR3/DR4 groups (DR4, HLA-DRB1*04-DQA1*03-DQB1*0302; DR3, HLA-DRB1*0301-DQA1*0501-DQB1*0201; DRX, HLA haplotype that is neither HLA-DRB1*04-DQA1*03-DQB1*0302 nor HLA-DRB1*0301-DQA1*0501-DQB1*0201). Characteristics are at enrolment except where noted otherwise

^a^The category ‘unknown’ represents those who reported their race as unknown, rather than missing data

^b^Hispanic/Latino is shown separately because race and ethnicity were collected as two different sets of classifications in the USA (until 2024)

Values that were statistically significant in the age-adjusted model are indicated by asterisks: *p<0.05; **p<0.01; ***p <0.001

T1D, type 1 diabetes

Progressors carrying DR3/non-DR4 had a higher prevalence of GADA (91.2%) and a lower prevalence of IA-2A (48.1%) and IAA (41.2%) than those with DR4/non-DR3 (77.2%, 69.5% and 55.0%, respectively,) or DR3/DR4 (83.3%, 68.8% and 56.5%; all comparisons, *p*<0.01) (Table 2). DR3/non-DR4 participants were significantly older at enrolment (mean 13.64 years) than those in the DR3/DR4 group (9.80 years, *p*<0.001). In addition, the racial distribution was significantly different between DR3/non-DR4 participants (with a lower proportion of White participants, 82.0%) compared with the DR4/non-DR3 (89.1%, *p*=0.004) and DR3/DR4 (91.8%, *p*<0.001) groups. DR4/non-DR3 progressors were older at enrolment and had a lower T1D-GRS2 (overall and HLA component) than DR3/DR4 progressors (Table 2). There were no statistical differences for the non-HLA component of the T1D-GRS2 between the four genetic groups of progressors.

### Other analyses

#### Analyses excluding participants with the protective HLA-DR2-DQB1*06:02 HLA allele

After excluding participants with the protective DR2-DQB1*06:02 HLA allele, the previous results were unchanged for all autoantibody-positive participants (ESM Table 1) as well as for the subset of progressors (ESM Table 2).

#### Single autoantibody-positive participants

We separately analysed participants who were positive for a single autoantibody at baseline (*n*=3997) (ESM Table 3). Similar to the findings in the overall cohort, IA-2A% was lower in the DRX/DRX group than in the DR4/non-DR3 (*p*<0.001) and DR3/DR4 (*p*<0.001) group, but similar to that in the DR3/non-DR4 group. The DRX/DRX group had the highest prevalence of IAA% (all comparisons, *p*<0.001), the lowest prevalence of dysglycaemia at baseline (all comparisons, *p*<0.01) and the lowest risk of progression to diabetes (all comparisons, *p*<0.001). Participants with DR3/DR4 had the highest Index60 (all comparisons, *p*<0.001), were the most likely to be siblings of an individual with type 1 diabetes (all comparisons, *p*<0.001) and had the highest risk of progression to diabetes (all comparisons, *p*<0.01). The T1D-GRS2 followed the same pattern as in the overall group, with all comparisons being statistically significant (*p*<0.001).

Among the participants who were single autoantibody-positive at baseline and progressed to diabetes (*n*=387) (ESM Table 4), the DR3/non-DR4 group had the highest prevalence of GADA and the lowest of IA-2A (all comparisons, *p*<0.001), and the DR3/DR4 group had the highest T1D-GRS2 (all comparisons, *p*<0.001). Although the patterns were similar to those observed in the overall group, many of the differences did not reach statistical significance.

#### Multiple autoantibody-positive participants

Among the participants who were positive for multiple autoantibodies at baseline (*n*=2507) (ESM Table 5), IA-2A% was significantly lower in the DRX/DRX and DR3/non-DR4 groups (and not different between these two groups) compared with the DR4-/non-DR3 and DR3/DR4 groups (and not different between these two groups) (all significant comparisons, *p*<0.001). The DRX/DRX group had the highest prevalence of IAA% (all comparisons, *p*<0.001), the lowest Index60 (all comparisons, *p*<0.01), the lowest prevalence of dysglycaemia (*p*<0.001) and the lowest progression to diabetes (*p*<0.01). None of these parameters were different among the other three genetic groups. Participants with DR3/DR4 had the youngest age at baseline (all comparisons, *p*<0.001). Similar to the findings in the overall cohort, the T1D-GRS2 was highest in the DR3/DR4 group and progressively lower in the DR4/non-DR3, DR3/non-DR4 and DRX/DRX groups (all comparisons, *p*<0.001).

Among the participants who were multiple autoantibody-positive at baseline and progressed to diabetes (*n*=905) (ESM Table 6), there were fewer significant differences among the groups. The prevalence of GADA was higher and that of IA2-A was lower in the DR3/non-DR4 group than in the DR4/non-DR3 group (*p*<0.001). The ages at enrolment and at diagnosis of diabetes were significantly lower in DR3/DR4 participants than in the DR4/non-DR3 and DRX/DRX groups (all comparisons, *p*<0.001). The T1D-GRS2 followed the usual pattern, and all differences were significant (*p*<0.001) except between the DR3/non-DR4 and DR4/non-DR3 groups.

#### Comparison of HLA-DR4-DQ8 and HLA-DR4-nonDQ8

In the overall cohort of autoantibody-positive individuals, participants with HLA-DR4-DQ8 (*n*=3603), compared with participants with HLA-DR4 but not HLA-DQ8 (*n*=381), had a significantly higher prevalence of IA-2A, lower prevalence of IAA, higher T1D-GRS2, higher risk of progression to clinical diabetes and higher prevalence of dysglycaemia at baseline (all comparisons, *p*<0.01). Among the progressors, participants with HLA-DR4-DQ8 (*n*=890), compared with those with DR4-non-DQ8 (*n*=64), had a significantly higher T1D-GRS2 (*p*<0.001), but other variables were not significantly different.

#### Haplotypes in the DRX/DRX group

A total of 171 unique haplotypes were included in the DRX/DRX group (ESM Table 7).

## Discussion

To our knowledge, this is the first study to systematically assess islet autoantibodies, metabolic measures and demographic characteristics in paediatric and adult autoantibody-positive relatives without high-risk HLA haplotypes. We hypothesised that the absence of HLA-DRB1*04-DQA1*03-DQB1*0302 (HLA-DR4-DQ8) and/or HLA-DRB1*0301-DQA1*0501-DQB1*0201 (HLA-DR3-DQ2) (the two highest-risk HLA haplotypes) indicates phenotypic differences among initially non-diabetic but autoantibody-positive relatives. Our results indicate that those lacking both HLA-DR3-DQ2 and HLA-DR4-DQ8 had a lower prevalence of GADA and IA-2A positivity, older age at baseline, lower prevalence of White participants, higher prevalence of overweight/obesity, lower Index60 and lower 2 h glucose compared with participants who carried both risk haplotypes (DR3/DR4). Comparisons with the DR4/non-DR3 group followed a similar pattern, as DRX/DRX participants had a lower frequency of IA-2A and lower Index60 and 2 h glucose values, with a reduced prevalence of White or non-Hispanic/Latino participants. In contrast, compared with DR3/non-DR4 participants, the DRX/DRX group had a similar prevalence of IA-2A, higher prevalence of IAA, younger age and higher prevalence of Hispanic participants. Differences between the four genetic groups were observed even when restricting the analysis to the participants who tested positive for multiple autoantibodies at baseline and progressed to diabetes on follow-up.

Risk factors for islet autoimmunity in individuals who lack the two highest-risk HLA haplotypes (DR4-DQ8 and DR3-DQ2) may include low- or moderate-risk HLA haplotypes, or possibly other HLA haplotypes that have been associated with type 1 diabetes in adults and non-European populations [23–29]. In our study, among the 171 unique haplotypes that were included in the DRX/DRX group, three have been reported to be associated with increased type 1 diabetes risk: One of these, HLA-DRB1*08:01-DQA1*04:01-DQB1*04:02 (with a haplotype frequency in the DRX/DRX group of 4%) has a moderate effect on type 1 diabetes risk (OR 1.81) [30]. The other two risk haplotypes have been identified in African ancestry populations [25, 27] and were rare in the DRX/DRX group (0.08% for HLA-DRB1*07:01-DQA1*03:01-DQB1*02:01 and 0.54% for HLA-DRB1*09:01-DQA1*03:01-DQB1*02:01). Overall, and in the subset who progressed to clinical type 1 diabetes, the T1D-GRS2 was highest in the DR3/DR4 group and lowest in the DRX/DRX group, which is expected because of the strong weight of DR4-DQ8 and DR3-DQ2 and their interaction term in calculation of the T1D-GRS2 [21]. These findings suggest that genetic factors such as the type 1 diabetes-associated HLA haplotypes (and therefore the T1D-GRS2), which were defined using cohorts of mostly European-ancestry individuals with paediatric-onset type 1 diabetes, may underestimate the risk for this disease in older individuals of non-European ancestry. This has important implications for the design of strategies to screen for type 1 diabetes in the general population [31].

Additional risk factors for the development of islet autoimmunity in DRX/DRX individuals may include variants in non-HLA genetic regions and/or environmental causes. Research is needed to better understand these influences and whether the pathogenic mechanisms are different to those in DR3/DR4 individuals. In our study, consistent with our hypothesis, participants in the DRX/DRX group had characteristics that are atypical for paediatric type 1 diabetes, such as a lower Index60, than each of the other HLA groups, and more frequent overweight/obesity and older age than the DR3/DR4 group. These results are consistent with analyses of individuals who were recently diagnosed with type 1 diabetes, which found that lower-risk HLA genotypes are associated with older age at diagnosis, longer duration of symptoms before clinical onset (suggesting slower preclinical progression) and a lower prevalence of multiple islet autoantibody positivity [32–36]. Although the number of individuals presenting with type 1 diabetes in adult life is greater than the number presenting in childhood [37], the pathogenesis of adult-onset type 1 diabetes is still not fully understood. Furthermore, the different clinical characteristics, compared with those that are typical in paediatric type 1 diabetes, increase the risk of misdiagnosis as type 2 diabetes. A potential plausible explanation for the effect of age on type 1 diabetes heterogeneity is that the pathogenesis of islet autoimmunity and type 1 diabetes in DRX/DRX individuals may involve factors that are classically associated with obesity and type 2 diabetes. Indeed, our previous study of individuals with type 1 diabetes found a higher prevalence of type 2 diabetes-associated *TCF7L2* SNPs in those who lacked high-risk HLA haplotypes [38].

In agreement with previous data showing that IA-2A positivity indicates imminent progression to clinical type 1 diabetes [39], this autoantibody was most prevalent among DR3/DR4 participants, who had the highest type 1 diabetes risk by survival analysis and the lowest age at diagnosis of diabetes. It is uncertain whether this association is a consequence of the disease being at a more advanced stage at the time of screening due to faster disease progression in DR3/DR4 individuals or whether it represents a distinct pathway. The prevalence of IA-2A was lower in the DRX/DRX group compared with DR3/DR4 or DR4/non-DR3 individuals among autoantibody-positive relatives but not among the progressors, where it was similar. This pattern was not observed for DR3/non-DR4 participants, who had a lower prevalence of IA-2A compared with DR3/DR4 or DR4/non-DR3 participants in both the overall autoantibody-positive group and the subset who progressed to clinical type 1 diabetes. Furthermore, among participants who were single autoantibody-positive at screening and progressed to clinical type 1 diabetes on follow-up, the DR3/non-DR4 group had the lowest prevalence of IA-2A positivity among the four groups. A better understanding of distinct pathophysiological mechanisms that lead to type 1 diabetes [40] could direct therapies for treatment or prevention and enhance precision medicine for this disease [41, 42].

Emerging data indicate that the HLA-DR4-DQ8 haplotype is more frequent in young children who develop IAA as the first autoantibody and progress more rapidly to clinical type 1 diabetes (before the age of 7 years), with nearly complete destruction of beta cells in the pancreas, strong lymphocytic infiltration and an elevated ratio of proinsulin to C-peptide (a marker of beta cell stress) [43–49]. In contrast, the HLA-DR3-DQ2 haplotype has been shown to be associated with a type 1 diabetes phenotype that is characterised by older age at diagnosis, GADA as the first islet autoantibody to appear and the most prevalent islet autoantibody, and features that are consistent with milder insulitis and greater residual beta cell mass [43–49]. Our analysis, which, to our knowledge, is unique in not excluding participants with low-risk HLA haplotypes, demonstrated that DR3/non-DR4 participants had the lowest prevalence of IAA positivity and the oldest age at enrolment of the four genetic groups. DR3/non-DR4 participants also had a lower prevalence of IA-2A, a higher prevalence of overweight/obesity and a higher prevalence of African-American participants than the DR4/non-DR3 and DR3/DR4 participants (with these three characteristics being similar to those in the DRX/DRX group). The prevalence of GADA was higher in DR3/non-DR4 participants than in DR4/non-DR3 participants. Moreover, among the progressors, we observed a higher prevalence of GADA, and a lower prevalence of IA-2A and IAA in DR3/non-DR4 participants than in each of the other three groups. Among the progressors who were single-positive at screening, the DR3/non-DR4 group had the lowest prevalence of IA-2A and the highest prevalence of GADA. Thus, regarding IA-2A and IAA, overall, DR3/non-DR4 participants actually differed from the other groups more than DRX/DRX did. The observed lower prevalence of IA-2A and IAA in the DR3/non-DR4 group compared with the other groups was unexpected, and we do not have an explanation. In contrast, we observed fewer differences between the DR4/non-DR3 and DR3/DR4 groups. The comparison between autoantibody-positive participants who carried HLA-DR4-DQ8 and those with HLA-DR4 but not HLA-DQ8 demonstrated that most features that are associated with DR3/DR4 (e.g. prevalence of IA-2A positivity, dysglycaemia, T1D-GRS2 and risk of progression to type 1 diabetes) were significantly higher in participants with HLA-DR4-DQ8, except for IAA positivity, which was significantly more prevalent in those with HLA-DR4 but not HLA-DQ8. Overall, these data suggest differences in the pathogenesis of islet autoimmunity and type 1 diabetes in individuals with HLA-DR3-DQ2 compared with other HLA risk factors.

One limitation of this study was the possibility of a ‘survivor effect’, in which subsets of individuals who progress rapidly through preclinical stages may be under-represented in the TrialNet Pathway to Prevention cohort. TrialNet enrols relatives of individuals with type 1 diabetes, and thus participants have an increased genetic background risk for the disease. It should be noted that participants are identified as autoantibody-positive at the time of enrolment into the study, but the time of seroconversion is not known, and the longitudinal course of autoantibody positivity has not been studied. On the other hand, the cross-sectional study design, in which individuals of any age without diabetes are enrolled and tested for islet autoantibodies, may render our results more generalisable to type 1 diabetes risk screening in the general population [50]. Another limitation is the predominant representation of non-Hispanic White participants, but the large sample size in this cohort allowed us to identify some differences among other races and ethnicities. More studies on understudied races and ethnic groups in the USA and worldwide, as well as studies in the general population, are necessary. There may be additional differences between our study sample and the general population in terms of sex/gender and regional or socioeconomic factors that could limit the generalisability of our findings. A strength of the study was that TrialNet does not select participants for specific type 1 diabetes-associated genetic factors. Thus, we were able to evaluate low-risk HLA genotypes that had not been analysed by previous studies [43, 44, 51, 52]. The effect of sex was analysed and found not significant. The availability of metabolic data and the inclusion of participants across the life span, including adolescence and adulthood, are additional strengths of the study.

In conclusion, autoantibody-positive individuals who lack the two highest-risk HLA DR4-DQ8 and DR3-DQ2 haplotypes are more likely to be older and of non-European ancestry compared with those who have them, and thus may be missed by screening programmes that use these haplotypes or T1D-GRS2, in which DR4-DQ8 and DR3-DQ2 have strong weight. The more atypical characteristics, including greater insulin secretion in relation to glucose (as reflected by a lower Index60) in DRX/DRX compared with the other genetic groups, suggest additional underlying mechanisms, possibly related to insulin resistance or other type 2 diabetes-related pathogenesis. Finally, we found further evidence that DR3/non-DR4 individuals may follow a distinct pathogenic pathway compared with other genetic groups, including those with low-risk HLA haplotypes. Altogether, these findings appear consistent with the concept that a variety of genetic predispositions lead to unique pathogenic mechanisms that coalesce under a common clinical presentation of type 1 diabetes. Furthermore, these results may inform ongoing and future screening programmes for type 1 diabetes risk that incorporate HLA genotyping of participants who test positive for islet autoantibodies.

## Supplementary Information

Below is the link to the electronic supplementary material.

ESM (PDF 656 KB)

## Data Availability

The datasets generated and analysed during the current study are available from the Type 1 Diabetes TrialNet Coordinating Center on reasonable request.

## Abbreviations

DR3/DR4: Carrying *HLA-DRB1*04-DQA1*03-DQB1*0302* (HLA-DR4-DQ8) and *HLA-DRB1*0301-DQA1*0501-DQB1*0201* (HLA-DR3-DQ2)
DR4/non-DR3: Carrying HLA-DR4-DQ8 but not HLA-DR3-DQ2
DR3/non-DR4: Carrying HLA-DR3-DQ2 but not HLA-DR4-DQ8
DRX/DRX: Carrying neither HLA-DR4-DQ8 nor HLA-DR3-DQ2
T1D-GRS2: Type 1 diabetes genetic risk score 2
