# Supplementary material for: Characteristics of autoantibody-positive individuals without high-risk HLA-DR4-DQ8 or HLA-DR3-DQ2 haplotypes
Source: Diabetologia. 2024 Dec 13;68(3):588–601. doi: 10.1007/s00125-024-06338-7 (PMC11832693; doi:10.1007/s00125-024-06338-7)
Supplement: Supplementary file 1 — ESM (PDF 656 KB) [file 125_2024_6338_MOESM1_ESM.pdf]

ELECTRONIC SUPPLEMENTARY MATERIAL (ESM)

ESM Table 1. Characteristics of autoantibody-positive relatives excluding those with the protective haplotype HLA-DR2-DQB*06:02: Comparisons between HLA DRX/DRX, DR3/non-DR4, DR4/non-DR3 and DR3/DR4 (DR4: HLA-DRB1*04-DQA1*03-DQB1*0302, DR3: HLA-DRB1*0301-DQA1*0501-DQB1*0201, DRX: HLA haplotype that is neither HLA-DRB1*04-DQA1*03-DQB1*0302 nor HLA-DRB1*0301-DQA1*0501-DQB1*0201).

Parameter		DRX/DRX (A) N=1125	DR3/non-DR4 (B) N=1496	DR4/non-DR3 (C) N=2226	DR3/DR4 (D) N=1263	Overall p-value	p-value (A vs. B)	p-value (A vs. C)	p-value (A vs. D)	p-value (B vs. C)	p-value (B vs. D)	p-value (C vs. D)
GADA Status - % (N)	GAD-	23.7 (267)	15.8 (237)	20.4 (454)	15.5 (196)	<0.001*	<0.001*	0.026	<0.001*	<0.001#	0.816	<0.001*
	GAD+	76.3 (858)	84.2 (1259)	79.6 (1772)	84.5 (1067)							
ICA512 Status - % (N)	ICA512-	77.6 (873)	79.5 (1190)	60.3 (1342)	55.2 (697)	<0.001*	0.228	<0.001*	<0.001*	<0.001*	<0.001*	0.003
	ICA512+	22.4 (252)	20.5 (306)	39.7 (884)	44.8 (566)							
IAA Status - % (N)	IAA-	55.2 (621)	69.4 (1038)	58.2 (1296)	59.0 (745)	<0.001*	<0.001*	0.095	0.062*	<0.001*	<0.001#	0.659
	IAA+	44.8 (504)	30.6 (458)	41.8 (930)	41.0 (518)							
Age (yrs) at Autoantibody Draw - Mean (SD, N)		17.04 (12.65, 1125)	18.14 (13.61, 1494)	16.61 (12.72, 2224)	13.96 (11.30, 1263)	<0.001	0.034	0.359	<0.001	<0.001	<0.001	<0.001
Gender - % (N)	Female	52.0 (584)	52.6 (786)	51.4 (1144)	48.9 (617)	0.244	0.740	0.767	0.135	0.475	0.052	0.152
	Male	48.0 (540)	47.4 (708)	48.6 (1081)	51.1 (645)							

Parameter		DRX/DRX (A) N=1125	DR3/non-DR4 (B) N=1496	DR4/non-DR3 (C) N=2226	DR3/DR4 (D) N=1263	Overall p-value	p-value (A vs. B)	p-value (A vs. C)	p-value (A vs. D)	p-value (B vs. C)	p-value (B vs. D)	p-value (C vs. D)
Race - % (N)	Asian	2.7 (30)	2.3 (34)	0.9 (20)	1.0 (13)	<0.001 *	0.592	<0.001*	<0.001*	<0.001*	<0.001*	0.696
	Black or African American	4.5 (50)	4.7 (70)	2.4 (53)	1.8 (23)							
	More than One Race	2.1 (23)	1.6 (23)	1.7 (38)	1.2 (15)							
	White	82.9 (918)	84.7 (1251)	87.8 (1928)	89.1 (1110)							
	Refused	0.5 (5)	0.2 (3)	0.2 (5)	0.2 (3)							
	Unknown or not reported	7.4 (82)	6.5 (96)	6.9 (152)	6.6 (82)							
Hispanic or Latino - % (N)	Yes	14.1 (157)	7.8 (116)	10.4 (228)	10.1 (127)	<0.001 *	<0.001*	0.005#	0.011	0.026	0.102	0.913
	No	82.1 (916)	87.9 (1307)	85.0 (1872)	85.5 (1074)							
	Unknown	3.9 (43)	4.3 (64)	4.6 (102)	4.4 (55)							
Overweight or Obese - % (N)	BMI < 25	75.6 (826)	73.3 (1063)	77.9 (1693)	80.1 (981)	<0.001 *	0.183	0.152	0.010	0.002	<0.001	0.132
	BMI >= 25	24.4 (266)	26.7 (387)	22.1 (481)	19.9 (244)							

Parameter		DRX/DRX (A) N=1125	DR3/non-DR4 (B) N=1496	DR4/non-DR3 (C) N=2226	DR3/DR4 (D) N=1263	Overall p-value	p-value (A vs. B)	p-value (A vs. C)	p-value (A vs. D)	p-value (B vs. C)	p-value (B vs. D)	p-value (C vs. D)
Baseline Index60 - Mean (SD, N)		-0.13 (1.31, 1125)	0.04 (1.38, 1496)	0.13 (1.46, 2226)	0.38 (1.38, 1263)	<0.001 *	0.001*	<0.001*	<0.001*	0.083	<0.001*	<0.001*
T1D-GRS2 – Mean (SD, N)		11.41 (1.85, 692)	13.11 (1.59, 929)	13.70 (1.36, 1528)	15.38 (1.12, 920)	<0.001 *	<0.001*	<0.001*	<0.001*	<0.001*	<0.001*	<0.001*
HLA T1D-GRS2 – Mean (SD, N)		7.46 (1.58, 692)	9.14 (1.37, 929)	9.69 (1.15, 1528)	11.47 (0.83, 920)	<0.001 *	<0.001*	<0.001*	<0.001*	<0.001*	<0.001*	<0.001*
Non-HLA T1D-GRS2 – Mean (SD, N)		3.98 (0.80, 692)	4.00 (0.84, 929)	4.02 (0.78, 1528)	3.92 (0.78, 920)	0.031@	0.743	0.312	0.121	0.491	0.046	0.003#
Relative With Diabetes - % (N)	Father	9.4 (106)	10.8 (162)	11.7 (260)	8.1 (102)	<0.001 *	<0.001#	0.004#	<0.001*	<0.001	<0.001*	<0.001*
	Mother	8.1 (91)	8.0 (119)	7.8 (173)	8.2 (104)							
	Offspring	19.1 (215)	23.8 (356)	17.8 (396)	11.9 (150)							
	Sibling	50.3 (566)	49.6 (742)	53.4 (1189)	64.4 (814)							
	Other	13.1 (147)	7.8 (117)	9.3 (208)	7.4 (93)							
T1D Progressor - % (N)	No	87.9 (989)	83.0 (1241)	77.2 (1718)	70.5 (891)	<0.001 *	<0.001*	<0.001*	<0.001*	<0.001*	<0.001*	<0.001#
	Yes	12.1 (136)	17.0 (255)	22.8 (508)	29.5 (372)							
Follow-up Time (yrs) - Mean (STD, N)		2.39 (1.94, 1125)	2.27 (1.94, 1496)	2.39 (1.90, 2226)	2.35 (1.86, 1263)	0.291	0.138	0.978	0.613	0.072	0.308	0.536

Parameter		DRX/DRX (A) N=1125	DR3/non-DR4 (B) N=1496	DR4/non-DR3 (C) N=2226	DR3/DR4 (D) N=1263	Overall p-value	p-value (A vs. B)	p-value (A vs. C)	p-value (A vs. D)	p-value (B vs. C)	p-value (B vs. D)	p-value (C vs. D)
Fasting Glucose (mmol/L)- Mean (STD, N)		5.11 (0.69, 1125)	5.16 (0.72, 1496)	5.18 (1.20, 2226)	5.09 (0.72, 1263)	0.024	0.079	0.073	0.507	0.555	0.013	0.017
2 hr Glucose (mmol/L)- Mean (STD, N)		6.64 (2.54, 1125)	6.91 (2.59, 1496)	7.11 (2.93, 2226)	7.25 (2.92, 126)	<0.001 *	0.009	<0.001*	<0.001*	0.027	<0.001*	0.172
Dysglycemia - % (N)	No	79.3 (892)	72.9 (1091)	70.4 (1568)	67.5 (852)	<0.001 *	<0.001*	<0.001*	<0.001*	0.100	0.002*	0.066
	Yes	20.7 (233)	27.1 (405)	29.6 (658)	32.5 (411)							
HbA1c mmol/mol- Mean (STD, N)		32.37 (0.42, 1102)	32.66 (0.41, 1467)	32.66 (0.43, 2166)	32.74 (0.38, 1238)	0.076@	0.052	0.133	0.009#	0.556	0.467	0.184
HbA1c %- Mean (STD, N)		5.11 (0.04, 1102)	5.14 (0.04, 1467)	5.14 (0.04, 2166)	5.15 (0.03, 1238)	0.076@	0.052	0.133	0.009#	0.556	0.467	0.184

Characteristics are at enrollment except where noted otherwise. Denominators for calculation of the percentages are not the number at the top of the column due to missing data.

* Age adjusted model p-value <0.001 (*), p-value <0.05 (@), p-value <0.01 (#)

ESM Table 2: Characteristics of autoantibody-positive relatives who progressed to clinical type 1 diabetes ("progressors") excluding those with the protective haplotype HLA-DR2-DQB*06:02: Comparisons between HLA DRX/DRX, DR3/non-DR4, DR4/non-DR3 and DR3/DR4 ((DR4: HLA-DRB1*04-DQA1*03-DQB1*0302, DR3: HLA-DRB1*0301-DQA1*0501-DQB1*0201, DRX: HLA haplotype that is neither HLA-DRB1*04-DQA1*03-DQB1*0302 nor HLA-DRB1*0301-DQA1*0501-DQB1*0201).

Parameter		DRX/DRX (A) A N=136	DR3/non-DR4 (D) B N=255	DR4/non-DR3 (C) C N=508	DR3/DR4 (B) D N=372	Overall p-value	p-value (A vs. B)	p-value (A vs. C)	p-value (A vs. D)	p-value (B vs. C)	p-value (B vs. D)	p-value (C vs. D)
GADA - % (N)	Neg	21.3 (29)	8.2 (21)	22.8 (116)	16.7 (62)	<0.001 *	<0.001#	0.708	0.226	<0.001*	0.002	0.024#
	Pos	78.7 (107)	91.8 (234)	77.2 (392)	83.3 (310)							
IA-2A - % (N)	Neg	35.3 (48)	51.8 (132)	30.3 (154)	31.2 (116)	<0.001 *	0.002#	0.266	0.380	<0.001*	<0.001*	0.783
	Pos	64.7 (88)	48.2 (123)	69.7 (354)	68.8 (256)							
IAA - % (N)	Neg	40.4 (55)	58.8 (150)	44.7 (227)	43.5 (162)	<0.001 @	<0.001#	0.376	0.531	<0.001#	<0.001	0.737
	Pos	59.6 (81)	41.2 (105)	55.3 (281)	56.5 (210)							
Age (yrs) at Autoantibody Draw - Mean (SD, N)		13.28 (10.56, 136)	13.54 (11.34, 255)	12.39 (10.22, 508)	9.80 (7.31, 372)	<0.001	0.826	0.368	<0.001	0.157	<0.001	<0.001

Parameter		DRX/DRX (A) A N=136	DR3/non-DR4 (D) B N=255	DR4/non-DR3 (C) C N=508	DR3/DR4 (B) D N=372	Overall p-value	p-value (A vs. B)	p-value (A vs. C)	p-value (A vs. D)	p-value (B vs. C)	p-value (B vs. D)	p-value (C vs. D)
Gender - % (N)	Female	50.7 (69)	47.8 (122)	51.4 (261)	44.2 (164)	0.192	0.586	0.894	0.191	0.357	0.369	0.036
	Male	49.3 (67)	52.2 (133)	48.6 (247)	55.8 (207)							
Race - % (N)	Asian	0.7 (1)	2.8 (7)	0.6 (3)	0.5 (2)	0.002	0.027	0.423	0.848	0.003	0.002	0.726
	Black or African American	2.2 (3)	6.0 (15)	1.8 (9)	1.6 (6)							
	Multiracial	0.7 (1)	1.6 (4)	2.0 (10)	0.8 (3)							
	White	91.8 (123)	82.1 (206)	89.3 (451)	91.8 (335)							
	Refused	1.5 (2)	0.0 (0)	0.4 (2)	0.5 (2)							
	Unknown or not reported	3.0 (4)	7.6 (19)	5.9 (30)	4.7 (17)							
Hispanic or Latino - % (N)	Yes	8.1 (11)	7.1 (18)	8.3 (42)	11.1 (41)	0.338	0.691	0.716	0.582	0.831	0.082	0.106
	No	88.2 (120)	87.4 (222)	86.3 (434)	85.9 (318)							
	Unknown	3.7 (5)	5.5 (14)	5.4 (27)	3.0 (11)							
Overweight or Obese - % (N)	BMI < 25	86.2 (112)	84.3 (210)	84.8 (413)	86.1 (310)	0.911@	0.639	0.701	0.990	0.868	0.542	0.595
	BMI >= 25	13.8 (18)	15.7 (39)	15.2 (74)	13.9 (50)							
Baseline Index60 - Mean (SD, N)		1.48 (1.40, 136)	1.42 (1.34, 255)	1.38 (1.51, 508)	1.33 (1.34, 372)	0.735	0.671	0.504	0.277	0.759	0.428	0.596

Parameter		DRX/DRX (A)<br>A<br>N=136	DR3/non-DR4 (D)<br>B<br>N=255	DR4/non-DR3 (C)<br>C<br>N=508	DR3/DR4 (B)<br>D<br>N=372	Overall p-value	p-value (A vs. B)	p-value (A vs. C)	p-value (A vs. D)	p-value (B vs. C)	p-value (B vs. D)	p-value (C vs. D)
T1D-GRS – Mean (SD, N)		12.51 (1.42, 105)	13.54 (1.47, 207)	13.94 (1.37, 405)	15.56 (1.12, 302)	<0.001 *	<0.001*	<0.001*	<0.001*	<0.001#	<0.001*	<0.001*
HLA T1D-GRS2 – Mean (SD, N)		8.29 (1.16, 105)	9.48 (1.36, 207)	9.88 (1.13, 405)	11.56 (0.84, 302)	<0.001 *	<0.001*	<0.001*	<0.001*	<0.001#	<0.001*	<0.001*
Non-HLA T1D-GRS2 – Mean (SD, N)		4.22 (0.73, 105)	4.07 (0.78, 207)	4.06 (0.75, 405)	4.02 (0.78, 302)	0.124	0.091	0.042	0.018	0.857	0.463	0.493
Relative With Diabetes - % (N)	Father	14.7 (20)	9.8 (25)	13.4 (68)	8.1 (30)	<0.001 @	0.061	0.600	0.008	0.026	0.001	<0.001#
	Mother	13.2 (18)	6.3 (16)	9.8 (50)	8.6 (32)							
	Offspring	8.1 (11)	12.2 (31)	7.9 (40)	3.8 (14)							
	Sibling	56.6 (77)	64.3 (164)	57.9 (294)	71.8 (267)							
	Other	7.4 (10)	7.5 (19)	11.0 (56)	7.8 (29)							
Age (yrs) at T1D Diagnosis - Mean (STD, N)		15.36 (10.47, 136)	15.61 (11.35, 255)	14.49 (10.13, 508)	12.01 (7.40, 372)	<0.001	0.827	0.381	<0.001	0.167	<0.001	<0.001
Time to T1D Diagnosis (yrs) - Mean (STD, N)		1.60 (1.40, 136)	1.48 (1.36, 255)	1.65 (1.50, 508)	1.76 (1.46, 372)	0.134	0.416	0.729	0.280	0.132	0.017	0.291
Fasting Glucose(mmol/L) - Mean (STD, N)		5.40 (1.50, 136)	5.51 (1.20, 255)	5.39 (1.41, 508)	5.20 (1.06, 372)	0.024	0.447	0.933	0.092	0.256	<0.001	0.030

Parameter		DRX/DRX (A) A N=136	DR3/non-DR4 (D) B N=255	DR4/non-DR3 (C) C N=508	DR3/DR4 (B) D N=372	Overall p-value	p-value (A vs. B)	p-value (A vs. C)	p-value (A vs. D)	p-value (B vs. C)	p-value (B vs. D)	p-value (C vs. D)
2 hr Glucose (mmol/L)- Mean (STD, N)		9.72 (4.79, 136)	9.66 (4.10, 255)	9.47 (4.54, 508)	9.13 (4.13, 372)	0.384	0.897	0.577	0.176	0.579	0.117	0.257
Dysglycemia - % (N)	No	44.1 (60)	35.3 (90)	39.6 (201)	43.5 (162)	0.156	0.087	0.337	0.909	0.252	0.038	0.236
	Yes	55.9 (76)	64.7 (165)	60.4 (307)	56.5 (210)							
HbA1c mmol/mol- Mean (STD, N)		35.51 (0.76, 134)	35.98 (0.57, 250)	35.17 (0.62, 494)	34.42 (0.49, 365)	0.028	0.515	0.701	0.095	0.138	<0.001	0.062
HbA1c %- Mean (STD, N)		5.40 (0.07, 134)	5.44 (0.05, 250)	5.37 (0.06, 494)	5.30 (0.04, 365)	0.028	0.515	0.701	0.095	0.138	<0.001	0.062

Characteristics are at enrollment except where noted otherwise. Denominators for calculation of the percentages are not the number at the top of the column due to missing data.

* Age adjusted model p-value <0.001 (*), p-value <0.05 (@), p-value <0.01 (#)

ESM Table 3: Characteristics of single autoantibody-positive relatives: Comparisons between HLA DRX/DRX, DR3/non-DR4, DR4/non-DR3 and DR3/DR4 ((DR4: HLA-DRB1*04-DQA1*03-DQB1*0302, DR3: HLA-DRB1*0301-DQA1*0501-DQB1*0201, DRX: HLA haplotype that is neither HLA-DRB1*04-DQA1*03-DQB1*0302 nor HLA-DRB1*0301-DQA1*0501-DQB1*0201).

Parameter		Not DR3 or DR4/DR8 (A) N=900	DR3 (B) N=1163	DR4/DR8 (C) N=1316	Both DR3 and DR4/DR8 (D) N=618	Overall p-value	p-value (A vs. B)	p-value (A vs. C)	p-value (A vs. D)	p-value (B vs. C)	p-value (B vs. D)	p-value (C vs. D)
GADA Status - % (N)	GAD-	32.9 (296)	21.8 (253)	30.0 (395)	26.4 (163)	<0.001	<0.001	0.152	0.007	<0.001	0.028	0.099
	GAD+	67.1 (604)	78.2 (910)	70.0 (921)	73.6 (455)							
ICA512 Status - % (N)	ICA512-	95.6 (860)	95.0 (1105)	92.0 (1211)	89.5 (553)	<0.001	0.566	<0.001	<0.001	0.003	<0.001	0.066
	ICA512+	4.4 (40)	5.0 (58)	8.0 (105)	10.5 (65)							
IAA Status - % (N)	IAA-	71.6 (644)	83.2 (968)	78.0 (1026)	84.1 (520)	<0.001	<0.001	<0.001	<0.001	<0.001	0.622	0.002
	IAA+	28.4 (256)	16.8 (195)	22.0 (290)	15.9 (98)							
Age (yrs) at AB Draw - Mean (STD, N)		19.04 (13.37, 900)	20.76 (14.12, 1162)	19.98 (13.98, 1314)	17.70 (13.25, 618)	<0.001	0.005	0.112	0.054	0.171	<0.001	<0.001
Gender - % (N)	Female	53.8 (484)	55.6 (646)	54.9 (722)	53.7 (332)	0.805	0.399	0.601	0.983	0.713	0.438	0.626
	Male	46.2 (416)	44.4 (515)	45.1 (593)	46.3 (286)							
Race - % (N)	Asian	2.8 (25)	2.3 (27)	0.9 (11)	2.1 (13)	<0.001	0.439	<0.001	<0.001	0.004	0.006	0.014

Parameter		Not DR3 or DR4/DR8 (A) N=900	DR3 (B) N=1163	DR4/DR8 (C) N=1316	Both DR3 and DR4/DR8 (D) N=618	Overall p-value	p-value (A vs. B)	p-value (A vs. C)	p-value (A vs. D)	p-value (B vs. C)	p-value (B vs. D)	p-value (C vs. D)
	Black or African American	4.1 (36)	4.2 (48)	2.2 (29)	1.2 (7)							
	More than One Race	2.3 (20)	1.5 (17)	1.6 (21)	0.5 (3)							
	White	82.1 (729)	85.1 (979)	87.2 (1128)	90.0 (546)							
	Refused	0.3 (3)	0.3 (3)	0.2 (3)	0.2 (1)							
	Unknown or not reported	8.4 (75)	6.7 (77)	7.8 (101)	6.1 (37)							
Hispanic or Latino - %(N)	No	80.5 (718)	88.1 (1019)	83.4 (1084)	88.0 (541)	<0.001	<0.001	0.052	<0.001	<0.001	0.852	0.024
	Unknown	4.0 (36)	4.5 (52)	4.6 (60)	4.1 (25)							
	Yes	15.5 (138)	7.4 (86)	11.9 (155)	8.0 (49)							
Overweight or Obese - % (N)	BMI < 25	70.6 (617)	67.4 (758)	71.4 (919)	76.0 (459)	0.002	0.124	0.704	0.022	0.034	<0.001	0.034
	BMI >= 25	29.4 (257)	32.6 (367)	28.6 (369)	24.0 (145)							
Baseline Index60 - Mean (STD, N)		-0.40 (1.21, 900)	-0.20 (1.30, 1163)	-0.29 (1.33, 1316)	-0.02 (1.39, 618)	<0.001	<0.001	0.044	<0.001	0.079	0.006	<0.001
T1D-GRS2 - Mean (STD, N)		10.58 (2.06, 488)	12.60 (2.05, 648)	13.39 (1.51, 802)	15.26 (1.12, 418)	<0.001	<0.001	<0.001	<0.001	<0.001	<0.001	<0.001

Parameter		Not DR3 or DR4/DR8 (A) N=900	DR3 (B) N=1163	DR4/DR8 (C) N=1316	Both DR3 and DR4/DR8 (D) N=618	Overall p-value	p-value (A vs. B)	p-value (A vs. C)	p-value (A vs. D)	p-value (B vs. C)	p-value (B vs. D)	p-value (C vs. D)
HLA T1D-GRS2 - Mean (STD, N)		6.74 (1.82, 488)	8.65 (1.86, 648)	9.42 (1.32, 802)	11.41 (0.81, 418)	<0.001	<0.001	<0.001	<0.001	<0.001	<0.001	<0.001
Non-HLA T1D-GRS2 - Mean (STD, N)		3.89 (0.84, 488)	3.98 (0.86, 648)	3.97 (0.78, 802)	3.86 (0.79, 418)	0.054	0.095	0.100	0.591	0.829	0.028	0.026
Relative With Diabetes - % (N)	Father	7.3 (66)	10.7 (125)	11.4 (150)	7.9 (49)	<0.001	<0.001	<0.001	<0.001	0.146	<0.001	<0.001
	Mother	8.0 (72)	9.2 (107)	7.5 (99)	7.6 (47)							
	Offspring	24.0 (216)	29.7 (345)	27.1 (356)	19.9 (123)							
	Sibling	45.0 (405)	42.6 (496)	44.4 (584)	57.9 (358)							
	Other	15.7 (141)	7.7 (90)	9.7 (127)	6.6 (41)							
T1D Progressor - % (N)	No	95.7 (861)	91.1 (1059)	88.9 (1170)	84.1 (520)	<0.001	<0.001	<0.001	<0.001	0.076	<0.001	0.003
	Yes	4.3 (39)	8.9 (104)	11.1 (146)	15.9 (98)							
Follow-up Time (yrs) - Mean (STD, N)		2.45 (1.94, 900)	2.32 (1.97, 1163)	2.50 (1.91, 1316)	2.54 (1.91, 618)	0.071	0.157	0.515	0.376	0.023	0.029	0.706
Fasting Glucose (mmol/L) - Mean (STD, N)		5.12 (0.69, 900)	5.17 (0.66, 1163)	5.18 (1.39, 1316)	5.12 (0.78, 618)	0.365	0.084	0.211	0.939	0.804	0.165	0.318

Parameter		Not DR3 or DR4/DR8 (A) N=900	DR3 (B) N=1163	DR4/DR8 (C) N=1316	Both DR3 and DR4/DR8 (D) N=618	Overall p-value	p-value (A vs. B)	p-value (A vs. C)	p-value (A vs. D)	p-value (B vs. C)	p-value (B vs. D)	p-value (C vs. D)
2 hr Glucose (mmol/L) - Mean (STD, N)		6.38 (2.18, 900)	6.67 (2.40, 1163)	6.62 (2.28, 1316)	6.84 (2.79, 618)	0.002	0.005	0.016	<0.001	0.574	0.190	0.067
Dysglycemia - % (N)	No	82.4 (742)	77.6 (902)	77.4 (1019)	75.2 (465)	0.004	0.006	0.004	<0.001	0.940	0.271	0.288
	Yes	17.6 (158)	22.4 (261)	22.6 (297)	24.8 (153)							
HbA1c (mmol/mol) - Mean (STD, N)		32.26 (0.42, 878)	32.45 (0.39, 1138)	32.08 (0.36, 1270)	32.45 (0.34, 605)	0.127	0.359	0.323	0.412	0.029	0.988	0.055
HbA1c (%) - Mean (STD, N)		5.1 (0.04, 878)	5.1 (0.03, 1138)	5.1 (0.03, 1270)	5.1 (0.03, 605)	0.127	0.359	0.323	0.412	0.029	0.988	0.055
Multiple autoantibody positivity -% (N)	No	100.0 (900)	100.0 (1163)	100.0 (1316)	100.0 (618)	1.000	1.000	1.000	1.000	1.000	1.000	1.000

Characteristics are at enrollment except where noted otherwise. Denominators for calculation of the percentages are not the number at the top of the column due to missing data.

* Age adjusted model p-value <0.001 (*), p-value <0.05 (@), p-value <0.01 (#)

ESM Table 4: Characteristics of single autoantibody-positive relatives who progressed to clinical type 1 diabetes ("progressors"): Comparisons between HLA DRX/DRX, DR3/non-DR4, DR4/non-DR3 and DR3/DR4 ((DR4: HLA-DRB1*04-DQA1*03-DQB1*0302, DR3: HLA-DRB1*0301-DQA1*0501-DQB1*0201, DRX: HLA haplotype that is neither HLA-DRB1*04-DQA1*03-DQB1*0302 nor HLA-DRB1*0301-DQA1*0501-DQB1*0201).

Parameter		Not DR3 or DR4/DR8 (A) N=39	DR3 (B) N=104	DR4/DR8 (C) N=146	Both DR3 and DR4/DR8 (D) N=98	Overall p-value	p-value (A vs. B)	p-value (A vs. C)	p-value (A vs. D)	p-value (B vs. C)	p-value (B vs. D)	p-value (C vs. D)
GADA Status - % (N)	GAD-	59.0 (23)	18.3 (19)	48.6 (71)	44.9 (44)	<0.001	<0.001	0.251	0.137	<0.001	<0.001	0.567
	GAD+	41.0 (16)	81.7 (85)	51.4 (75)	55.1 (54)							
ICA512 Status - % (N)	ICA512-	64.1 (25)	90.4 (94)	71.2 (104)	71.4 (70)	<0.001	<0.001	0.389	0.401	<0.001	<0.001	0.974
	ICA512+	35.9 (14)	9.6 (10)	28.8 (42)	28.6 (28)							
IAA Status - % (N)	IAA-	76.9 (30)	91.3 (95)	80.1 (117)	83.7 (82)	0.068	0.043	0.659	0.356	0.015	0.098	0.485
	IAA+	23.1 (9)	8.7 (9)	19.9 (29)	16.3 (16)							
Age (yrs) at AB Draw - Mean (STD, N)		15.70 (12.63, 39)	17.41 (13.15, 104)	15.43 (12.84, 146)	11.43 (9.53, 98)	0.005	0.486	0.908	0.033	0.236	<0.001	0.009
Gender - % (N)	Female	46.2 (18)	51.9 (54)	50.0 (73)	38.8 (38)	0.241	0.539	0.670	0.428	0.764	0.061	0.084
	Male	53.8 (21)	48.1 (50)	50.0 (73)	61.2 (60)							
Race - % (N)	Asian	0.0 (0)	2.9 (3)	0.7 (1)	2.1 (2)	0.307	0.454	0.739	0.729	0.285	0.204	0.250

Parameter		Not DR3 or DR4/DR8 (A) N=39	DR3 (B) N=104	DR4/DR8 (C) N=146	Both DR3 and DR4/DR8 (D) N=98	Overall p-value	p-value (A vs. B)	p-value (A vs. C)	p-value (A vs. D)	p-value (B vs. C)	p-value (B vs. D)	p-value (C vs. D)
	Black or African American	0.0 (0)	3.9 (4)	1.4 (2)	0.0 (0)							
	More than One Race	0.0 (0)	1.0 (1)	2.8 (4)	0.0 (0)							
	White	94.9 (37)	84.5 (87)	89.7 (130)	92.6 (88)							
	Refused	0.0 (0)	0.0 (0)	0.0 (0)	1.1 (1)							
	Unknown or not reported	5.1 (2)	7.8 (8)	5.5 (8)	4.2 (4)							
Hispanic or Latino - %(N)	No	87.2 (34)	87.5 (91)	84.7 (122)	87.6 (85)	0.600	0.868	0.699	0.821	0.153	0.339	0.808
	Unknown	5.1 (2)	6.7 (7)	3.5 (5)	3.1 (3)							
	Yes	7.7 (3)	5.8 (6)	11.8 (17)	9.3 (9)							
Overweight or Obese - % (N)	BMI < 25	81.1 (30)	74.5 (73)	78.1 (107)	88.5 (85)	0.086	0.422	0.694	0.260	0.519	0.012	0.039
	BMI >= 25	18.9 (7)	25.5 (25)	21.9 (30)	11.5 (11)							
Baseline Index60 - Mean (STD, N)		1.67 (1.63, 39)	1.41 (1.52, 104)	1.00 (1.68, 146)	1.25 (1.58, 98)	0.066	0.378	0.027	0.162	0.047	0.444	0.253
T1D-GRS2 - Mean (STD, N)		12.20 (1.44, 29)	13.22 (2.03, 81)	13.91 (1.33, 124)	15.40 (1.13, 84)	<0.001	0.014	<0.001	<0.001	0.004	<0.001	<0.001
HLA T1D-GRS2 - Mean (STD, N)		8.11 (1.31, 29)	9.12 (1.84, 81)	9.83 (1.13, 124)	11.44 (0.74, 84)	<0.001	0.008	<0.001	<0.001	<0.001	<0.001	<0.001

Parameter		Not DR3 or DR4/DR8 (A) N=39	DR3 (B) N=104	DR4/DR8 (C) N=146	Both DR3 and DR4/DR8 (D) N=98	Overall p-value	p-value (A vs. B)	p-value (A vs. C)	p-value (A vs. D)	p-value (B vs. C)	p-value (B vs. D)	p-value (C vs. D)
Non-HLA T1D-GRS2 - Mean (STD, N)		4.09 (0.81, 29)	4.10 (0.76, 81)	4.04 (0.79, 124)	3.96 (0.90, 84)	0.697	0.931	0.752	0.491	0.552	0.265	0.505
Relative With Diabetes - % (N)	Father	7.7 (3)	5.8 (6)	13.7 (20)	4.1 (4)	0.023	0.878	0.609	0.499	0.124	0.110	<0.001
	Mother	7.7 (3)	8.7 (9)	9.6 (14)	10.2 (10)							
	Offspring	12.8 (5)	18.3 (19)	17.8 (26)	6.1 (6)							
	Sibling	59.0 (23)	58.7 (61)	45.2 (66)	70.4 (69)							
	Other	12.8 (5)	8.7 (9)	13.7 (20)	9.2 (9)							
Age (yrs) at T1D Diagnosis - Mean (STD, N)		18.15 (12.38, 39)	19.58 (13.22, 104)	17.90 (12.69, 146)	13.99 (9.39, 98)	0.009	0.559	0.912	0.035	0.311	<0.001	0.010
Time to T1D Diagnosis (yrs) - Mean (STD, N)		1.51 (1.41, 39)	1.32 (1.38, 104)	1.74 (1.49, 146)	1.87 (1.50, 98)	0.043	0.478	0.392	0.202	0.027	0.008	0.508
Fasting Glucose (mmol/L) - Mean (STD, N)		5.64 (2.31, 39)	5.70 (1.29, 104)	5.43 (1.95, 146)	5.43 (1.52, 98)	0.589	0.859	0.569	0.521	0.231	0.173	0.974
2 hr Glucose (mmol/L) - Mean (STD, N)		10.96 (5.95, 39)	10.44 (4.59, 104)	9.14 (4.51, 146)	9.40 (5.01, 98)	0.061	0.581	0.038	0.122	0.026	0.126	0.671
Dysglycemia - % (N)	No	30.8 (12)	31.7 (33)	41.1 (60)	50.0 (49)	0.037	0.912	0.240	0.041	0.131	0.008	0.170
	Yes	69.2 (27)	68.3 (71)	58.9 (86)	50.0 (49)							

Parameter		Not DR3 or DR4/DR8 (A) N=39	DR3 (B) N=104	DR4/DR8 (C) N=146	Both DR3 and DR4/DR8 (D) N=98	Overall p-value	p-value (A vs. B)	p-value (A vs. C)	p-value (A vs. D)	p-value (B vs. C)	p-value (B vs. D)	p-value (C vs. D)
HbA1c (mmol/mol) - Mean (STD, N)		38.11 (1.22, 38)	36.64 (0.66, 103)	34.88 (0.57, 141)	34.56 (0.47, 95)	0.020	0.405	0.033	0.028	0.042	0.022	0.680
HbA1c (%) - Mean (STD, N)		5.6 (0.11, 38)	5.5 (0.06, 103)	5.3 (0.05, 141)	5.3 (0.04, 95)	0.020	0.405	0.033	0.028	0.042	0.022	0.680
Multiple autoantibody positivity - % (N)	No	100.0 (39)	100.0 (104)	100.0 (146)	100.0 (98)	1.000	1.000	1.000	1.000	1.000	1.000	1.000

Characteristics are at enrollment except where noted otherwise. Denominators for calculation of the percentages are not the number at the top of the column due to missing data.

* Age adjusted model p-value <0.001 (*), p-value <0.05 (@), p-value <0.01 (#)

ESM Table 5: Characteristics of multiple autoantibody-positive relatives: Comparisons between HLA DRX/DRX, DR3/non-DR4, DR4/non-DR3 and DR3/DR4 (DR4: HLA-DRB1*04-DQA1*03-DQB1*0302, DR3: HLA-DRB1*0301-DQA1*0501-DQB1*0201, DRX: HLA haplotype that is neither HLA-DRB1*04-DQA1*03-DQB1*0302 nor HLA-DRB1*0301-DQA1*0501-DQB1*0201).

Parameter		Not DR3 or DR4/DR8 (A) N=394	DR3 (B) N=444	DR4/DR8 (C) N=1024	Both DR3 and DR4/DR8 (D) N=645	Overall p-value	p-value (A vs. B)	p-value (A vs. C)	p-value (A vs. D)	p-value (B vs. C)	p-value (B vs. D)	p-value (C vs. D)
GADA Status - % (N)	GAD-	3.8 (15)	2.5 (11)	8.1 (83)	5.1 (33)	<0.001	0.268	0.004	0.329	<0.001	0.030	0.019
	GAD+	96.2 (379)	97.5 (433)	91.9 (941)	94.9 (612)							
ICA512 Status - % (N)	ICA512-	41.4 (163)	40.8 (181)	22.4 (229)	22.3 (144)	<0.001	0.859	<0.001	<0.001	<0.001	<0.001	0.986
	ICA512+	58.6 (231)	59.2 (263)	77.6 (795)	77.7 (501)							
IAA Status - % (N)	IAA-	22.3 (88)	35.1 (156)	34.5 (353)	34.9 (225)	<0.001	<0.001	<0.001	<0.001	0.806	0.932	0.864
	IAA+	77.7 (306)	64.9 (288)	65.5 (671)	65.1 (420)							
Age (yrs) at AB Draw - Mean (STD, N)		12.54 (9.49, 394)	12.52 (10.49, 443)	12.98 (10.11, 1024)	10.38 (7.46, 645)	<0.001	0.970	0.455	<0.001	0.422	<0.001	<0.001

Parameter		Not DR3 or DR4/DR8 (A) N=394	DR3 (B) N=444	DR4/DR8 (C) N=1024	Both DR3 and DR4/DR8 (D) N=645	Overall p-value	p-value (A vs. B)	p-value (A vs. C)	p-value (A vs. D)	p-value (B vs. C)	p-value (B vs. D)	p-value (C vs. D)
Gender - % (N)	Female	47.8 (188)	47.3 (210)	48.3 (494)	44.3 (285)	0.431	0.876	0.879	0.261	0.727	0.322	0.108
	Male	52.2 (205)	52.7 (234)	51.7 (529)	55.7 (359)							
Race - % (N)	Asian	1.8 (7)	1.6 (7)	0.9 (9)	0.0 (0)	0.018	0.696	0.093	0.002	0.087	0.002	0.241
	Black or African American	5.2 (20)	5.5 (24)	2.7 (27)	2.5 (16)							
	More than One Race	2.3 (9)	1.6 (7)	1.8 (18)	1.9 (12)							
	White	85.0 (329)	85.6 (374)	88.6 (898)	88.3 (564)							
	Refused	0.5 (2)	0.0 (0)	0.2 (2)	0.3 (2)							
	Unknown or not reported	5.2 (20)	5.7 (25)	5.9 (60)	7.0 (45)							
Hispanic or Latino - %(N)	No	87.0 (342)	88.6 (390)	87.1 (886)	83.2 (533)	0.069	0.721	0.596	0.241	0.268	0.042	0.022
	Unknown	3.8 (15)	3.0 (13)	4.8 (49)	4.7 (30)							
	Yes	9.2 (36)	8.4 (37)	8.1 (82)	12.2 (78)							
Overweight or Obese - % (N)	BMI < 25	86.1 (328)	85.5 (370)	84.5 (844)	84.1 (522)	0.807	0.795	0.456	0.384	0.640	0.537	0.818
	BMI >= 25	13.9 (53)	14.5 (63)	15.5 (155)	15.9 (99)							

Parameter		Not DR3 or DR4/DR8 (A) N=394	DR3 (B) N=444	DR4/DR8 (C) N=1024	Both DR3 and DR4/DR8 (D) N=645	Overall p-value	p-value (A vs. B)	p-value (A vs. C)	p-value (A vs. D)	p-value (B vs. C)	p-value (B vs. D)	p-value (C vs. D)
Baseline Index60 - Mean (STD, N)		0.36 (1.32, 394)	0.60 (1.36, 444)	0.60 (1.45, 1024)	0.76 (1.26, 645)	<0.001	0.009	0.004	<0.001	0.980	0.052	0.023
T1D-GRS2 - Mean (STD, N)		11.79 (1.85, 304)	13.27 (1.48, 342)	13.78 (1.37, 794)	15.48 (1.11, 502)	<0.001	<0.001	<0.001	<0.001	<0.001	<0.001	<0.001
HLA T1D-GRS2 - Mean (STD, N)		7.69 (1.63, 304)	9.25 (1.38, 342)	9.73 (1.16, 794)	11.53 (0.85, 502)	<0.001	<0.001	<0.001	<0.001	<0.001	<0.001	<0.001
Non-HLA T1D-GRS2 - Mean (STD, N)		4.12 (0.80, 304)	4.04 (0.80, 342)	4.05 (0.78, 794)	3.97 (0.77, 502)	0.063	0.180	0.219	0.009	0.705	0.242	0.060
Relative With Diabetes - % (N)	Father	12.9 (51)	12.4 (55)	12.4 (127)	8.2 (53)	0.003	0.475	0.978	0.015	0.572	<0.001	<0.001
	Mother	8.4 (33)	6.3 (28)	7.8 (80)	8.8 (57)							
	Offspring	7.4 (29)	10.4 (46)	8.2 (84)	4.2 (27)							
	Sibling	62.9 (248)	63.3 (281)	63.0 (645)	70.7 (456)							
	Other	8.4 (33)	7.7 (34)	8.6 (88)	8.1 (52)							
T1D Progressor - % (N)	No	73.9 (291)	64.9 (288)	63.7 (652)	57.5 (371)	<0.001	0.005	<0.001	<0.001	0.662	0.015	0.012
	Yes	26.1 (103)	35.1 (156)	36.3 (372)	42.5 (274)							

Parameter		Not DR3 or DR4/DR8 (A) N=394	DR3 (B) N=444	DR4/DR8 (C) N=1024	Both DR3 and DR4/DR8 (D) N=645	Overall p-value	p-value (A vs. B)	p-value (A vs. C)	p-value (A vs. D)	p-value (B vs. C)	p-value (B vs. D)	p-value (C vs. D)
Follow-up Time (yrs) - Mean (STD, N)		2.24 (1.88, 394)	2.11 (1.85, 444)	2.25 (1.89, 1024)	2.16 (1.80, 645)	0.559	0.317	0.968	0.502	0.211	0.654	0.374
Fasting Glucose (mmol/L) - Mean (STD, N)		5.08 (0.81, 394)	5.11 (0.81, 444)	5.16 (0.83, 1024)	5.05 (0.65, 645)	0.055	0.563	0.120	0.552	0.351	0.179	0.007
2 hr Glucose (mmol/L) - Mean (STD, N)		7.07 (2.97, 394)	7.44 (2.84, 444)	7.66 (3.42, 1024)	7.65 (2.99, 645)	0.009	0.067	0.003	0.002	0.223	0.228	0.964
Dysglycemia - % (N)	No	74.4 (293)	63.1 (280)	62.7 (642)	60.0 (387)	<0.001	<0.001	<0.001	<0.001	0.893	0.308	0.270
	Yes	25.6 (101)	36.9 (164)	37.3 (382)	40.0 (258)							
HbA1c (mmol/mol) - Mean (STD, N)		32.48 (0.41, 389)	33.28 (0.42, 436)	33.24 (0.48, 1003)	33.21 (0.41, 633)	0.044	0.012	0.012	0.011	0.904	0.814	0.904
HbA1c (%) - Mean (STD, N)		5.1 (0.04, 389)	5.2 (0.04, 436)	5.2 (0.04, 1003)	5.2 (0.04, 633)	0.044	0.012	0.012	0.011	0.904	0.814	0.904
Multiple autoantibody positivity - % (N)	Yes	100.0 (394)	100.0 (444)	100.0 (1024)	100.0 (645)	1.000	1.000	1.000	1.000	1.000	1.000	1.000

Characteristics are at enrollment except where noted otherwise. Denominators for calculation of the percentages are not the number at the top of the column due to missing data.

* Age adjusted model p-value <0.001 (*), p-value <0.05 (@), p-value <0.01 (#)

ESM Table 6: Characteristics of multiple autoantibody-positive relatives who progressed to clinical type 1 diabetes ("progressors"): Comparisons between HLA DRX/DRX, DR3/non-DR4, DR4/non-DR3 and DR3/DR4 (DR4: HLA-DRB1*04-DQA1*03-DQB1*0302, DR3: HLA-DRB1*0301-DQA1*0501-DQB1*0201, DRX: HLA haplotype that is neither HLA-DRB1*04-DQA1*03-DQB1*0302 nor HLA-DRB1*0301-DQA1*0501-DQB1*0201).

Parameter		Not DR3 or DR4/DR8 (A) N=103	DR3 (B) N=156	DR4/DR8 (C) N=372	Both DR3 and DR4/DR8 (D) N=274	Overall p-value	p-value (A vs. B)	p-value (A vs. C)	p-value (A vs. D)	p-value (B vs. C)	p-value (B vs. D)	p-value (C vs. D)
GADA Status - % (N)	GAD-	6.8 (7)	2.6 (4)	12.6 (47)	6.6 (18)	<0.001	0.120	0.099	0.937	<0.001	0.070	0.011
	GAD+	93.2 (96)	97.4 (152)	87.4 (325)	93.4 (256)							
ICA512 Status - % (N)	ICA512-	23.3 (24)	26.3 (41)	14.5 (54)	16.8 (46)	0.006	0.588	0.033	0.147	0.001	0.018	0.430
	ICA512+	76.7 (79)	73.7 (115)	85.5 (318)	83.2 (228)							
IAA Status - % (N)	IAA-	28.2 (29)	37.2 (58)	31.2 (116)	29.2 (80)	0.314	0.132	0.555	0.842	0.181	0.088	0.587
	IAA+	71.8 (74)	62.8 (98)	68.8 (256)	70.8 (194)							
Age (yrs) at AB Draw - Mean (STD, N)		12.29 (9.56, 103)	11.12 (9.21, 156)	11.44 (8.95, 372)	9.22 (6.25, 274)	0.002	0.328	0.402	<0.001	0.715	0.011	<0.001
Gender - % (N)	Female	51.5 (53)	45.5 (71)	51.9 (193)	46.2 (126)	0.372	0.349	0.939	0.359	0.182	0.898	0.151
	Male	48.5 (50)	54.5 (85)	48.1 (179)	53.8 (147)							

Parameter		Not DR3 or DR4/DR8 (A) N=103	DR3 (B) N=156	DR4/DR8 (C) N=372	Both DR3 and DR4/DR8 (D) N=274	Overall p-value	p-value (A vs. B)	p-value (A vs. C)	p-value (A vs. D)	p-value (B vs. C)	p-value (B vs. D)	p-value (C vs. D)
Race - % (N)	Asian	1.0 (1)	2.6 (4)	0.5 (2)	0.0 (0)	0.012	0.056	0.375	0.246	0.017	0.004	0.772
	Black or African American	3.0 (3)	7.2 (11)	2.2 (8)	2.2 (6)							
	More than One Race	1.0 (1)	2.0 (3)	1.6 (6)	1.1 (3)							
	White	91.1 (92)	80.4 (123)	88.9 (328)	91.5 (247)							
	Refused	2.0 (2)	0.0 (0)	0.5 (2)	0.4 (1)							
	Unknown or not reported	2.0 (2)	7.8 (12)	6.2 (23)	4.8 (13)							
Hispanic or Latino - %(N)	No	89.3 (92)	86.5 (134)	86.7 (320)	85.3 (233)	0.195	0.746	0.421	0.539	0.569	0.499	0.025
	Unknown	2.9 (3)	4.5 (7)	6.2 (23)	2.9 (8)							
	Yes	7.8 (8)	9.0 (14)	7.0 (26)	11.7 (32)							
Overweight or Obese - % (N)	BMI < 25	87.9 (87)	89.0 (138)	85.8 (309)	85.2 (225)	0.680	0.778	0.600	0.517	0.325	0.269	0.831
	BMI >= 25	12.1 (12)	11.0 (17)	14.2 (51)	14.8 (39)							
Baseline Index60 - Mean (STD, N)		1.41 (1.36, 103)	1.40 (1.22, 156)	1.51 (1.40, 372)	1.36 (1.24, 274)	0.528	0.945	0.522	0.735	0.391	0.755	0.161

Parameter		Not DR3 or DR4/DR8 (A) N=103	DR3 (B) N=156	DR4/DR8 (C) N=372	Both DR3 and DR4/DR8 (D) N=274	Overall p-value	p-value (A vs. B)	p-value (A vs. C)	p-value (A vs. D)	p-value (B vs. C)	p-value (B vs. D)	p-value (C vs. D)
T1D-GRS2 - Mean (STD, N)		12.42 (1.60, 81)	13.54 (1.36, 131)	13.89 (1.44, 288)	15.62 (1.11, 218)	<0.001	<0.001	<0.001	<0.001	0.019	<0.001	<0.001
HLA T1D-GRS2 - Mean (STD, N)		8.15 (1.36, 81)	9.51 (1.30, 131)	9.83 (1.19, 288)	11.60 (0.88, 218)	<0.001	<0.001	<0.001	<0.001	0.012	<0.001	<0.001
Non-HLA T1D-GRS2 - Mean (STD, N)		4.27 (0.68, 81)	4.04 (0.78, 131)	4.06 (0.73, 288)	4.04 (0.72, 218)	0.081	0.029	0.021	0.012	0.783	0.993	0.731
Relative With Diabetes - % (N)	Father	16.5 (17)	13.5 (21)	12.9 (48)	9.5 (26)	0.049	0.094	0.331	0.028	0.229	0.093	0.137
	Mother	14.6 (15)	5.1 (8)	9.7 (36)	8.0 (22)							
	Offspring	5.8 (6)	7.7 (12)	4.8 (18)	2.9 (8)							
	Sibling	57.3 (59)	66.7 (104)	62.6 (233)	72.3 (198)							
	Other	5.8 (6)	7.1 (11)	9.9 (37)	7.3 (20)							
	Sibling	57.3 (59)	66.7 (104)	62.6 (233)	72.3 (198)							
Age (yrs) at T1D Diagnosis - Mean (STD, N)		14.23 (9.42, 103)	13.10 (9.15, 156)	13.39 (8.82, 372)	11.30 (6.42, 274)	0.003	0.336	0.399	<0.001	0.733	0.018	<0.001

Parameter		Not DR3 or DR4/DR8 (A) N=103	DR3 (B) N=156	DR4/DR8 (C) N=372	Both DR3 and DR4/DR8 (D) N=274	Overall p-value	p-value (A vs. B)	p-value (A vs. C)	p-value (A vs. D)	p-value (B vs. C)	p-value (B vs. D)	p-value (C vs. D)
Time to T1D Diagnosis (yrs) - Mean (STD, N)		1.66 (1.41, 103)	1.57 (1.33, 156)	1.61 (1.49, 372)	1.72 (1.44, 274)	0.713	0.622	0.760	0.713	0.797	0.298	0.342
Fasting Glucose (mmol/L) - Mean (STD, N)		5.38 (1.38, 103)	5.38 (1.11, 156)	5.37 (1.12, 372)	5.12 (0.82, 274)	0.013	0.987	0.923	0.025	0.886	0.005	0.002
2 hr Glucose (mmol/L) - Mean (STD, N)		9.22 (4.32, 103)	9.13 (3.65, 156)	9.58 (4.50, 372)	9.03 (3.76, 274)	0.375	0.859	0.474	0.679	0.276	0.791	0.105
Dysglycemia - % (N)	No	50.5 (52)	37.8 (59)	38.7 (144)	41.2 (113)	0.153	0.044	0.032	0.107	0.848	0.486	0.516
	Yes	49.5 (51)	62.2 (97)	61.3 (228)	58.8 (161)							
HbA1c (mmol/mol) - Mean (STD, N)		34.69 (0.57, 102)	35.52 (0.49, 152)	35.34 (0.63, 363)	34.37 (0.49, 270)	0.155	0.261	0.394	0.624	0.773	0.037	0.057
HbA1c (%) - Mean (STD, N)		5.3 (0.05, 102)	5.4 (0.04, 152)	5.4 (0.06, 363)	5.3 (0.04, 270)	0.155	0.261	0.394	0.624	0.773	0.037	0.057
Multiple autoantibody positivity - % (N)	Yes	100.0 (103)	100.0 (156)	100.0 (372)	100.0 (274)	1.000	1.000	1.000	1.000	1.000	1.000	1.000

Characteristics are at enrollment except where noted otherwise. Denominators for calculation of the percentages are not the number at the top of the column due to missing data.

* Age adjusted model p-value <0.001 (*), p-value <0.05 (@), p-value <0.01 (#)

ESM Table 7: HLA Haplotypes observed in the HLA-DRX/DRX group

Haplotype (DRB1$DQA1$DQB1)	Count	Frequency (%)
0701$0201$0202	369	14.27
0101$0101$0501	308	11.91
0401$0301$0301	243	9.40
1302$0102$0604	194	7.50
1501$0102$0602	153	5.92
1301$0103$0603	150	5.80
0801$0401$0402	108	4.18
0102$0101$0501	68	2.63
1601$0102$0502	61	2.36
1101$0501$0301	56	2.17
0701$0201$0303	49	1.89
1101$0505$0301	49	1.89
0401$0303$0301	48	1.86
0901$0301$0303	43	1.66
1401$0101$0503	34	1.31
1001$0101$0501	31	1.20
0103$0101$0501	29	1.12
1302$0102$0609	28	1.08
1201$0501$0301	20	0.77
1201$0505$0301	20	0.77
0407$0301$0301	18	0.70
1104$0501$0301	18	0.70
0802$0401$0402	17	0.66
1502$0103$0601	17	0.66
0302$0401$0402	16	0.62
1104$0505$0301	15	0.58
0408$0301$0304	14	0.54
0901$0301$0201	14	0.54
1303$0501$0301	14	0.54
1503$0102$0602	14	0.54
0405$0301$0201	13	0.50
0408$0301$0301	13	0.50
0401$0301$0304	10	0.39
1303$0505$0301	10	0.39
0901$0301$0202	9	0.35
1501$0102$0502	9	0.35
1602$0102$0502	9	0.35
1302$0102$0501	8	0.31
1404$0101$0503	8	0.31

1502$0102$0602	8	0.31
0405$0301$0202	7	0.27
1102$0501$0301	7	0.27
1103$0505$0301	7	0.27
0701$0201$0201	6	0.23
0804$0401$0402	6	0.23
0401$0301$0305	5	0.19
0403$0301$0304	5	0.19
0901$0302$0303	5	0.19
1103$0501$0301	5	0.19
1301$0102$0604	5	0.19
1303$0201$0202	5	0.19
0404$0301$0301	4	0.15
0405$0303$0202	4	0.15
0405$0505$0301	4	0.15
0408$0303$0301	4	0.15
0801$0301$0302	4	0.15
0803$0601$0301	4	0.15
1201$0101$0501	4	0.15
1301$0102$0501	4	0.15
1501$0102$0601	4	0.15
1501$0102$0603	4	0.15
1502$0102$0502	4	0.15
0103$0505$0301	3	0.12
0403$0301$0301	3	0.12
0405$0301$0401	3	0.12
0406$0301$0402	3	0.12
0801$0402$0402	3	0.12
0804$0501$0301	3	0.12
0804$0505$0301	3	0.12
1406$0503$0301	3	0.12
1602$0505$0301	3	0.12
0301$0102$0602	2	0.08
0305$0501$0201	2	0.08
0404$0301$0304	2	0.08
0405$0103$0503	2	0.08
0405$0301$0301	2	0.08
0405$0303$0401	2	0.08
0407$0303$0301	2	0.08
0408$0303$0304	2	0.08
0409$0301$0301	2	0.08
0701$0201$0301	2	0.08
0701$0301$0201	2	0.08

0701$0301$0202	2	0.08
0806$0102$0602	2	0.08
0811$0401$0402	2	0.08
1102$0505$0319	2	0.08
1104$0103$0603	2	0.08
1202$0601$0301	2	0.08
1301$0103$0608	2	0.08
1301$0301$0303	2	0.08
1302$0102$0502	2	0.08
1302$0102$0602	2	0.08
1302$0103$0603	2	0.08
1302$0301$0302	2	0.08
1304$0505$0319	2	0.08
1305$0501$0301	2	0.08
1305$0505$0301	2	0.08
1401$0101$0502	2	0.08
1402$0501$0301	2	0.08
1404$0101$0501	2	0.08
1406$0501$0301	2	0.08
1408$0101$0503	2	0.08
1501$0102$0620	2	0.08
1501$0401$0402	2	0.08
1502$0101$0501	2	0.08
1502$0103$0503	2	0.08
1506$0102$0502	2	0.08
1602$0501$0301	2	0.08
0101$0101$0502	1	0.04
0101$0101$0602	1	0.04
0101$0102$0504	1	0.04
0101$0103$0601	1	0.04
0103$0501$0301	1	0.04
0107$0101$0501	1	0.04
0301$0102$0501	1	0.04
0301$0103$0604	1	0.04
0302$0501$0203	1	0.04
0304$0501$0201	1	0.04
0315$0501$0201	1	0.04
0401$0301$0201	1	0.04
0401$0301$0311	1	0.04
0401$0301$0312	1	0.04
0403$0301$0305	1	0.04
0403$0301$0402	1	0.04
0404$0301$0305	1	0.04

0404$0301$0311	1	0.04
0405$0301$0402	1	0.04
0405$0501$0301	1	0.04
0406$0303$0402	1	0.04
0407$0301$0306	1	0.04
0410$0301$0402	1	0.04
0411$0301$0402	1	0.04
0601$0102$0502	1	0.04
0701$0101$0303	1	0.04
0701$0201$0302	1	0.04
0803$0103$0601	1	0.04
0804$0301$0201	1	0.04
0804$0401$0301	1	0.04
0805$0401$0402	1	0.04
0806$0505$0301	1	0.04
0901$0301$0302	1	0.04
0901$0303$0202	1	0.04
1101$0102$0502	1	0.04
1101$0102$0602	1	0.04
1101$0501$0201	1	0.04
1101$0501$0302	1	0.04
1101$0502$0301	1	0.04
1101$0505$0319	1	0.04
1102$0102$0502	1	0.04
1102$0102$0602	1	0.04
1104$0501$0201	1	0.04
1107$0501$0301	1	0.04
1201$0501$0302	1	0.04
1202$0501$0301	1	0.04
1301$0103$0501	1	0.04
1302$0101$0501	1	0.04
1302$0101$0503	1	0.04
1303$0301$0202	1	0.04
1304$0501$0301	1	0.04
1305$0501$0302	1	0.04
1306$0103$0603	1	0.04
1401$0101$0603	1	0.04
1402$0301$0302	1	0.04
1403$0501$0301	1	0.04
1501$0101$0501	1	0.04
1501$0103$0601	1	0.04
1501$0505$0301	1	0.04
1502$0102$0504	1	0.04

1502$0102$0601	1	0.04
1503$0301$0202	1	0.04
1602$0505$0302	1	0.04

ESM Figure 1. Flowchart that illustrates selection of participants for this analysis. Abbreviations: AB: Islet autoantibody; IAA: insulin autoantibodies, GADA: GAD65 autoantibodies, IA-2A: Insulinoma-associated antigen 2 autoantibodies. OGTT: Oral glucose tolerance test. DR4: HLA-DRB1*04-DQA1*03-DQB1*0302, DR3: HLA-DRB1*0301-DQA1*0501-DQB1*0201.

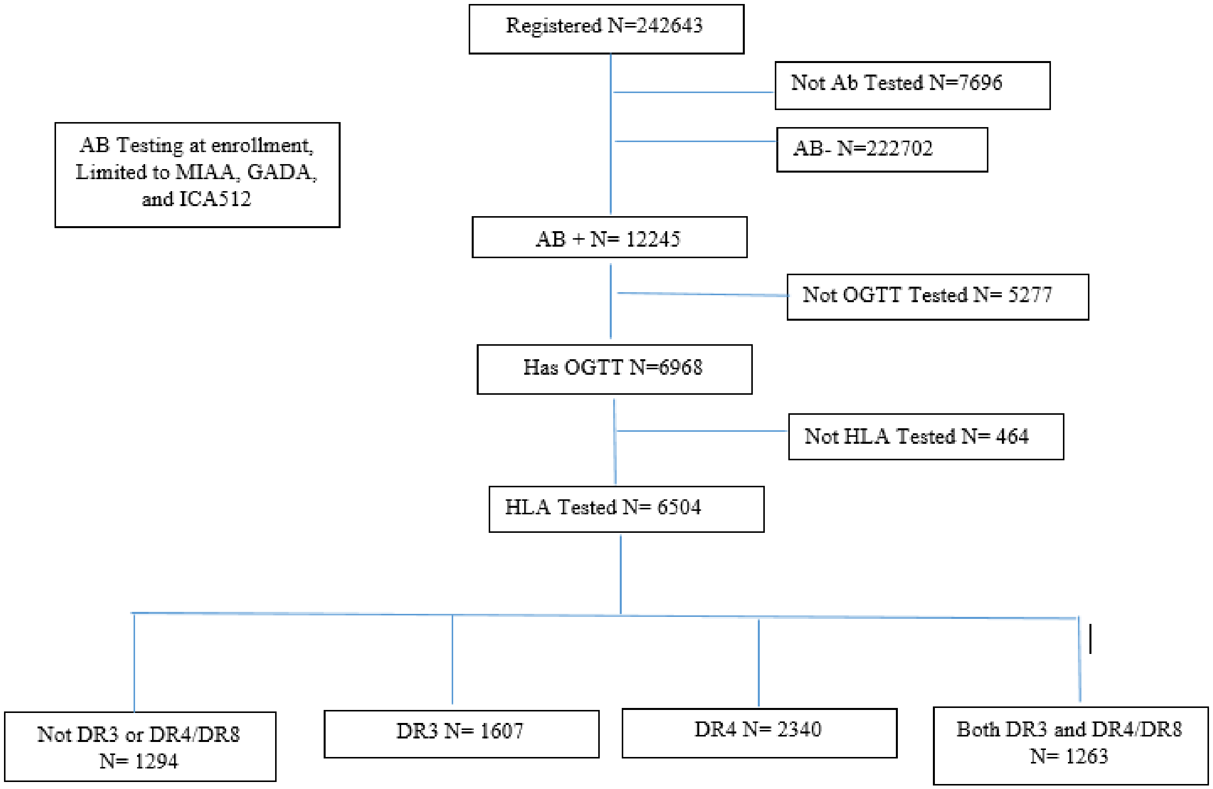

ESM Figure 2. Survival analysis of progression to clinical T1D in autoantibody positive relatives of individuals with T1D, by HLA group, DR4: HLA-DRB1*04-DQA1*03-DQB1*0302, DR3: HLA-DRB1*0301-DQA1*0501-DQB1*0201. DRX: HLA haplotype that is neither DR3-DQ2 nor DR4-DQ8)**. P-value <0.001**

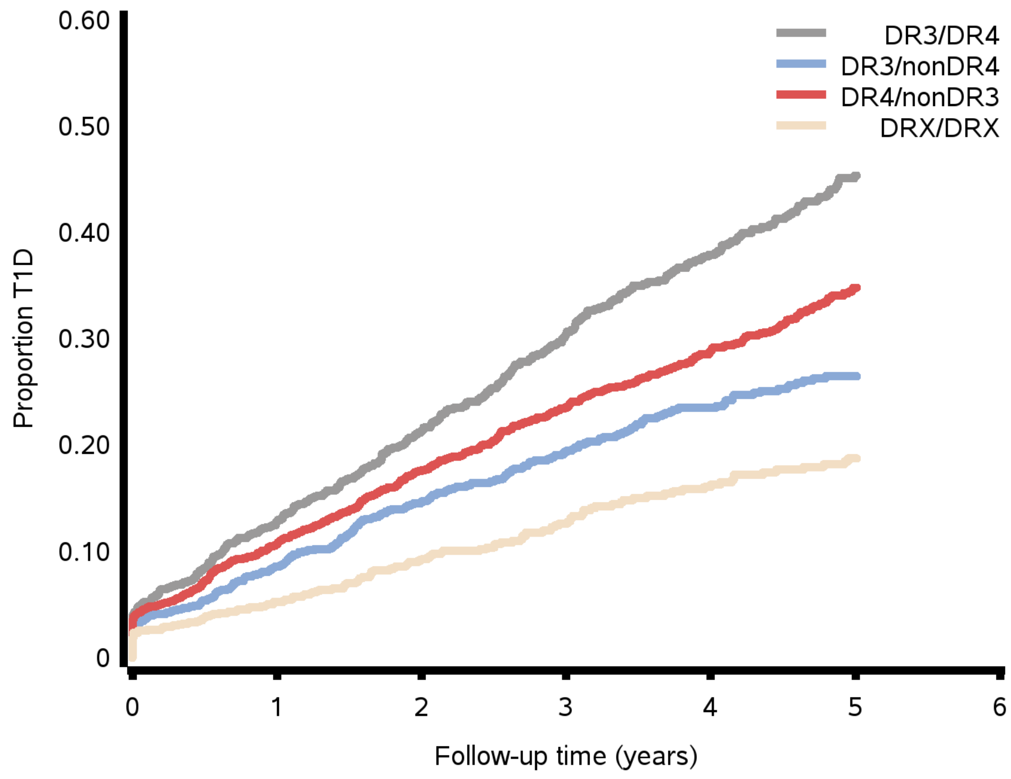

Number at risk:						
DR3/DR4	1263	850	657	476	347	240
DR3/nonDR4	1607	1037	776	600	449	349
DR4/nonDR3	2340	1599	1224	908	684	510
DRX/DRX	1294	871	678	502	374	294

List of Type 1 Diabetes TrialNet Study Group members as of 4/28/2023

Steering Committee: Kevan Herold (Yale University), Mark Anderson (University of California, San Francisco), Mark A. Atkinson (University of Florida), Todd Brusko (University of Florida), Jane Buckner (Benaroya Research Institute), Mark Clements (The Children's Mercy Hospital), Peter G. Colman (Walter & Eliza Hall Institute of Medical Research), Mark Daniels (Children's Hospital of Orange County), Linda DiMeglio (Indiana University), Carmella EvansMolina (Indiana University), Jason Gaglia (Joslin Diabetes Center), Stephen E. Gitelman (University of California, San Francisco), Robin Goland (Columbia University), Peter Gottlieb (Barbara Davis Center for Childhood Diabetes), Michael Haller (University of Florida), Carla J. Greenbaum (Benaroya Research Institute), Martin Hessner (Medical College of Wisconsin), Jeffrey P. Krischer (University of South Florida), Megan Levings (University of British Columbia), Ingrid Libman (University of Pittsburgh), Peter Linsley (Benaroya Research Institute), Alice Long (Benaroya Research Institute), Sandra Lord (Benaroya Research Institute), Wayne Moore (The Children's Mercy Hospital), Antoinette Moran (University of Minnesota), Andrew Muir (Emory Children's Center), Priya Pralahad (Stanford University), William Russell (Vanderbilt Eskind Diabetes Clinic), Jennifer Sherr (Yale University), Lisa Spain (National Institute of Diabetes and Digestive and Kidney Diseases [NIDDK]), Andrea Steck (Barbara Davis Center for Childhood Diabetes), John Wentworth (Walter & Eliza Hall Institute of Medical Research), Diane Wherrett (University of Toronto), Perrin White (University of Texas Southwestern), Darrell M. Wilson (Stanford University), William Winter (University of Florida). Past Members: Peter Antinozzi (Wake Forest University), David A. Baidal (University of Miami), Manuela Battaglia (San Raffaele University), Dorothy Becker (University of Pittsburgh), Penelope Bingley (University of Bristol), Emanuele Bosi (San Raffaele University), Richard Insel (JDRF), Thomas Kay (St. Vincent's Institute of Medical Research), Mikael Knip (University of Helsinki), Åke Lernmark (Skane University Hospital), Yuk-Fun Liu (University of Bristol), Jennifer B. Marks (University of Miami), Jerry Palmer (University of Washington), Mark Peakman (King's College), Louis Philipson (University of Chicago), Alberto Pugliese (University of Miami), Philip Raskin (University of Texas Southwestern), Maria Redondo (Baylor College of Medicine), Henry Rodriguez (University of South Florida Diabetes and Endocrinology Center), Bart Roep (Leiden University Medical Center), Desmond A. Schatz (University of Florida), Jay S. Skyler (University of Miami), Jay M. Sosenko (University of Miami), Jorma Toppari (Hospital District of Southwest Finland), Anette Ziegler (Technical University Munich). Executive Committee: Kevan Herold (Yale University), Linda DiMeglio (Indiana University), Carla J. Greenbaum (Benaroya Research Institute), Jeffrey P. Krischer (University of South Florida), Ellen Leschek (National Institute of Diabetes and Digestive and Kidney Diseases [NIDDK]), Lisa Spain (National Institute of Diabetes and Digestive and Kidney Diseases [NIDDK]). 2 Past Members: Katarzyna Bourcier (National Institute of Allergy and Infectious Diseases [NIAID]), Richard Insel (JDRF), John Ridge (National Institute of Allergy and Infectious Disease [NIAID]), Jay S. Skyler (University of Miami). Chair's Office: Kevan Herold, (Yale University), Lisa Rafkin (University of Miami). Past Members: Carla J. Greenbaum (Benaroya Research Institute), Irene Santiago (University of Miami), Jay S. Skyler (University of Miami), Jay M. Sosenko (University of Miami). TrialNet Coordinating Center (University of South Florida): Jeffrey P. Krischer, Brian Bundy, Michael Abbondondolo, Rajesh Adusumalli, Logan Alford, Matthew Boonstra, Jessica Conaty, David Cuthbertson, Julie Ford, Jennifer Garmeson, Veena Gowda, Cameron Hainline, Brian Hays, Kathleen Heyman, Christina Karges, Amy Kunz, Shu Liu, Kristin Maddox, Colleen Maguire, Margaret Moore, Sarah Muller, Melissa Murray, Johanna Nesbitt, Ryan O'Donnell, Melissa Parker, MJ Pereyra, Francisco Perez Laras, Aswani Raheja, Devon Rizzo, Ariana Rojas, Cintia Reichert, Lisa Steward, Michael Taylor, Roy Tamura, Dena Tewey, Elon Walker-Veras, Jianmei Wang, Melissa Wroble, Lili Wurmser, Lu You, Kenneth Young. Past Members: Timothy Adams, Darlene Amado, Ilma Asif, Jenna Bjellquist, Laura Bocchino, Cristina Burroughs, Mario Cleves,

Meagan DeSalvatore, Christopher Eberhard, Steve Fiske, Susan Geyer, Courtney Henderson, Martha Henry, Belinda Hsiao, Amanda Kinderman, BeataGabriela Koziol, Lindsay Lane, Ashley Leinbach, Jennifer Lloyd, Jamie Malloy, Julie Martin, Cameron McNeill, Jessica Miller, Thuy Nguyen, Jodie Nunez, Nichole Reed, Amy Roberts, Kelly Sadler, Tina Stavros, Christine Sullivan, Megan V. Warnock, Keith Wood, Rebecca Wood, Ping Xu, Vanessa Yanek. National Institute of Diabetes and Digestive and Kidney Diseases [NIDDK]: Ellen Leschek, Lisa Spain. Data Safety and Monitoring Board: Emily Blumberg (University of Pennsylvania), Sean Aas (Georgetown University), Gerald Beck (Cleveland Clinic Foundation), Rose Gubitosi-Klug (Case Western Reserve University), Dennis Wallace (Retired). Past Members: David Brillon (Cornell University), Lori Laffel (Joslin Diabetes Center), Robert Veatch (Georgetown University), Robert Vigersky (Medtronic). Infectious Disease Safety Committee: Brett Loechelt (Children's National Medical Center), Lindsey Baden (Brigham and Women's Hospital), Peter Gottlieb (Barbara Davis Center for Childhood Diabetes), Michael Green (University of Pittsburgh), Ellen Leschek (National Institute of Diabetes and Digestive and Kidney Diseases [NIDDK]), Ingrid Libman (University of Pittsburgh), Adriana Weinberg (University of Colorado), John Wentworth (Walter & Eliza Hall Institute of Medical Research). Past Members: Nora Bryant (Joslin Diabetes Center),Yuk-Fun Liu (University of Bristol). 3 Laboratory Directors: Michael Sheldon (Infinity BiologiX), Adriana Weinberg (University of Colorado), William Winter (University of Florida), Liping Yu (Barbara Davis Center for Childhood Diabetes). Past Members: Santica Marcovina (University of Washington), Jerry P. Palmer (University of Washington), Jay Tischfield (Rutgers University). Psychosocial Committee: Suzanne Bennett-Johnson (Florida State University), Ryan McDonough (Children's Mercy), Kelli Delallo (University of Pittsburgh), Krim Driscoll (University of Florida), Kevan Herold (Yale University), Christine March (University of Pittsburgh), Sarah McGaugh (Hospital for Sick Children Toronto), Arielle Pagryzinski (HUB), Lisa Rafkin (University of Miami), Jennifer Sherr (Yale University), Korey Hood (Stanford University), Diane Naranjo (Stanford University), Anna Barash (Benaroya), Holly O'Donnell (Barbara Davis Center), Brittany Bruggeman (University of Florida), Paige Trojanowski (Barbara Davis Center), Linda DiMeglio (Indiana University), Kali Johnson (University of Minnesota). TrialNet Clinical Network Hub (Benaroya Research Institute): Arielle Pagryzinski, Emily Batts, Danielle Tom, Catherine Nguyen, Chris Budy. Past Members: Annie Schultz, Kristin Fitzpatrick, Randy Guerra, Melita Romasco, Annie Shultz, Mary Ramey, Michele Patience-Staal, Meghan Tobin, Diana Skye, Christopher Webb. Underrepresented Minority Committee: Ananta Addala (Stanford University), Susanne Cabrera (University of California San Francisco), Kevan Herold (Yale University, Lisa Rafkin (University of Miami), Andy Muir (Emory University), Robin Goland (Columbia University), Darrell Wilson (Stanford University), Ingrid Libman (University of Pittsburgh), Wayne Moore (The Children's Mercy Hospital), Carla Greenbaum (Benaroya), Linda DiMeglio (Indiana University), Brittany Bruggeman (University of Florida). Active Personnel at Clinical Centers Participating in the TN01 Protocol: Barbara Davis Center for Childhood Diabetes, Aurora, Colorado: Andrea K. Steck, Lexie Chesshir, Peter A. Gottlieb, Lisa Meyers, Aaron W. Michels, Marian Rewers, Kimber Simmons, Morgan Sooy, Fatima Tensun, Taylor Triolo, Leah Galvez Valencia, Paula Wadwa, Ruthie Williamson. Benaroya Research Institute, Seattle, Washington: Carla J. Greenbaum, Jane H. Buckner, Sadiq El'Amin-White, Sandra Lord, Bao Ng, Mary Ramey, Michael Richter, Elaine Sachter, Corinna Tordillos, Dana VanBuecken, Kimberly Varner, Heather White, Nancy Wickstrom, Cassandra Williams, Alyssa Ylescupidez. The Children's Hospital of Orange County: Amrit Bhangoo, Mark Daniels, Daina Dreimane, Mark Daniels, Marissa Erickson, Timothy Flannery, Nikta Forghani, Sarah Hu, Himala Kashmiri, Anabel Palencia, Christina Reh, Francoise Sutton, Heather Speer, Lien Trihn. 4 The Children's Mercy Hospital, Kansas City, Missouri: Wayne Moore, Fadi Al Muhaisen, Jennifer Boyd, Julia Broussard, Mark Clements, Aliza Elrod, Katelyn Evans, Max Feldt, Kelsee Halpin, Heather Harding, Jennifer James, Terri Luetjen, Ryan McDonough, Susan Mitchell, Tiffany Musick, Emily Paprocki, Rhiannon Pomerantz, Nikita Raje, Luis Sainz y Diaz, Britaney

Spartz. Columbia University, New York, New York: Robin Goland, Mone Anzai, Magdalena Bogun, Rachelle Gandica, Natasha Leibel, Jacqueline Lonier, James Pring, Nathan Schwab, Kristen Williams. Emory Children's Center, Atlanta, Georgia: Andrew Muir, Amber Antich, Kristina Cossen, Eric Felner, Lynette Gonzalez, Wanda Sanchez, Catherine Simpson. The Hospital for Sick Children, Toronto, Ontario: Diane K. Wherrett, Lesley Eisel, Sarah McGaugh, Rebecca Stochinsky, Mary Jo Ricci. Indiana University, Indianapolis, Indiana: Linda A. DiMeglio, Carmella Evans-Molina, Jamie Felton, Heba M. Ismail, Megan Kirchner, Anna Neyman, Juan Sanchez, Corinne Parks-Schenck, Emily K. Sims, Maria Spall. Stanford University, Stanford, California: Darrell M. Wilson, Bonnie Baker, Karen Barahona, Bruce Buckingham, Priya Prahalad, Trudy Esrey. University of British Columbia, Kelowna, British Columbia: Constadina Panagiotopoulos, Daniel Metzger, Lauren Semkow. University of California, San Francisco, California: Stephen Gitelman, Fatema Abdulhussein, Natalie Aceves, Mark Anderson, Julissa Cabrera, Hannah Chessner, Abby Cobb-Walch, Laura Dapkus, Aristides Diamant, Marysol Gonzales Granados, Tina Hu, Karen Ko, Janet Lee, Roger Long, Isabella Niu, Srinath Sanda, Caroline Schulmeister, Priya Srivastava, Lorraine Stiehl, Christine Torok, Rebecca Wesch, Jenise Wong, Kevin Yen. University of Florida, Gainesville, Florida: Michael Haller, Mark Atkinson, Brittany Bruggeman, Todd M. Brusko, Miriam Cintron, Kristin Dayton, Timothy Foster, Jennifer Hosford, Laura Jacobsen, Sarah Peeling, Danielle Poulton, Desmond Schatz, Malinda Tran, William Winter. University of Minnesota, Minneapolis, Minnesota: Antoinette Moran, Shannon Beasley, Melena Bellin, Jane Kennedy, Janice Leschyshyn, Brandon Nathan, Beth Pappenfus, Ihsan Rizky, Muna Sunni. 5 University of Pittsburgh, Pittsburgh, Pennsylvania: Ingrid Libman, Dorothy Becker, Kelli DeLallo, Christine March, Carly Shelleby, Frederico Toledo. University of Texas Southwestern, Dallas, Texas: Perrin White, Abha Choudhary, Yasmin Dominguez, Philip Raskin, Serey Sao. Vanderbilt Eskind Diabetes Clinic, Nashville, Tennessee: William Russell, Faith Brendle, Justin Gregory, Brenna Hammel, Jenny Leshko, Daniel Moore, Kimberly Rainer, Tyler Smith. Walter and Eliza Hall Institute of Medical Research, Parkville, Victoria: Peter Colman, John M. Wentworth, Candice Breen, Marika Bjorasen, Spiros Fourlanos, Leonard Harrison, Felicity Healy, Leanne Redl. Yale University, New Haven, Connecticut: Jennifer Sherr, Kevan Herold, Lori Carria, Jeanine May, William Tamborlane, Eileen Tichy, Stuart Weinzimer, Kate Weyman.
